# A pH‐sensitive Macromolecular Prodrug as TLR7/8 Targeting Immune Response Modifier

**DOI:** 10.1002/chem.201702942

**Published:** 2017-09-04

**Authors:** Stefan Aichhorn, Anne Linhardt, Angela Halfmann, Markus Nadlinger, Stefanie Kirchberger, Manuela Stadler, Barbara Dillinger, Martin Distel, Alexander Dohnal, Ian Teasdale, Wolfgang Schöfberger

**Affiliations:** ^1^ Institute of Organic Chemistry Johannes Kepler University Altenberger Straße 69 4040 Linz Austria; ^2^ Institute of Polymer Chemistry Johannes Kepler University Altenberger Straße 69 4040 Linz Austria; ^3^ Tumorimmunology and Innovative Cancer Models, St. Anna Kinderkrebsforschung e.V. CCRI-Children's Cancer Research Institute Zimmermannplatz 10 1090 Vienna Austria

**Keywords:** imidazoquinoline, immunochemistry, nanovaccines, polymers, TLR7/8 agonists

## Abstract

The chemical synthesis and biological activity of novel functionalized imidazoquinoline derivatives (ImQ) to generate Toll‐like receptor (TLR) 7/8 specific prodrugs are presented. In vivo activity of ImQs to induce inflammation was confirmed in zebrafish larvae. After covalent ligation to fully biodegradable polyphosphazenes (ImQ‐polymer), the macromolecular prodrugs were designed to undergo intracellular pH‐sensitive release of ImQs to induce inflammation through binding to endosomal TLR7/8 (danger signal). We showed ImQ dissociation from prodrugs at a pH 5 pointing towards endosomal prodrug degradability. ImQ‐polymers strongly activated ovalbumin‐specific T cells in murine splenocytes as shown by increased proliferation and expression of the IL‐2 receptor (CD25) on CD8+ T cells accompanied by strong IFN‐γ release. ImQ prodrugs presented here are suggested to form the basis of novel nanovaccines, for example, for intravenous or intratumoral cancer immunotherapeutic applications to trigger physiological antitumor immune responses.

Agonists of toll‐like receptors (TLRs) are being actively pursued for their ability to activate the immune system for a host of therapeutic applications, including cancer immunotherapy.^1^ Murine TLR7^2^ and human TLRs 7 and 8^3^ are known to sense guanosine‐based drugs that induce an antiviral response in vivo, stimulating the development of tolerogenic antigen‐presenting dendritic cells (DCs), producing selective cytokines that drive T cell responses. To date, most DC‐based tumor immune‐therapeutic strategies involve ex vivo loading of DCs with tumor‐associated antigens and immune‐stimulatory agents (adjuvants) and subsequent re‐injection into the patient for in vivo T cell activation.^4^ This represents a highly promising but labor and cost intensive methodology. Thus, a more direct, pharmaceutical approach could be of significant value to the field.^5^ However, low molecular weight molecular adjuvants share a poor pharmaceutical profile and rapid diffusion from the local tissue, even via intratumoral delivery, and hence the risk of systemic immune response represents a severe limitation of the applicable dose to levels below that required for activation. One of the most widely investigated groups of TLR agonists are imidazoquinolines, a family of synthetic small molecules, including imiquimod (R837), resiquimod (R848), gardiquimod, and other variants. Herein we present a novel imidazo[4,5‐*c*]quinolin‐4‐amine agonist, chemically modified as such to enable reversible covalent ligation to synthetic and/or biological macromolecules, that is, macromolecular prodrugs. The rational for a macromolecular prodrug approach, alongside potential gains in therapeutic efficacy^6^, ^7^ is the location of the TLR7/8 receptors in the endosome, well understood to be the major intracellular gateway for macromolecules.^8^ During the last decade, polymer‐based nanomedicines or polymer therapeutics^9^, ^10^ have become an important tool for drug delivery^6^ and theranostics.^11^ Although there can be clinical issues with heterogeneity and reproducibility the use of macromolecular prodrugs has been shown in a wide number of cases to increase the efficacy and positively influencing the biodistribution of therapeutics.^6^, ^9^ Poly(organo)phosphazenes are a versatile class of polymers with immense potential for application in nanomedicine.^12^, ^13^ Recent advances in polyphosphazene synthesis allow simple access to controlled molecular weights, controlled hydrodynamic volumes and high water solubility.^14^ One of relatively few fully degradable,^13^, ^15^ water soluble polymers available, essential design features in avoiding the deleterious effects associated with post‐drug‐release accumulation of high molecular weight macromolecules in the organism.^7^ Moreover, certain poly(organo)phosphazenes themselves appear as potent immunoadjuvants in vaccine delivery in terms of their complexes with protein antigens leading to activation and antigen presentation of DCs.^16^ Recently, a pH releasing nanogel approach was successfully applied to induce superior antibody and T cell responses against a tuberculosis antigen.^17^ Herein, we demonstrate the design of macromolecular prodrugs, capable of undergoing a controlled intracellular release of the low‐molecular weight TLR agonist as a so‐called “danger signal” to induce inflammation through binding to endosomal TLR7/8. However, since known imidazoquinoline based agonists lack appropriate sites for conjugation to suitable pH release systems, the implementation of novel synthetic imidazoquinoline analogues is necessary. We report the synthesis of novel a imidazo[4,5‐c]quinoline derivative with an aliphatic ketone moiety attached to the benzylic group, which was suitable for coupling to macromolecules equipped with hydrazide linkers to give pH cleavable hydrazone linkage,^18^ well‐proven to provide a clean rapid release in the acidic endosomal environment, thus to potentially produce a controlled, intracellular presentation of the TLR agonist. The group of David et al. have investigated the structure–activity relationships of imidazoquinoline analogues.^19^

It was demonstrated that maintaining high agonistic activity of imidazoquinolines narrows the possibilities to introduce functional groups down to a few certain positions of the 1*H*‐imidazo[4,5‐*c*]quinoline active core. Most notably, position C4 is required to hold a primary amine and C2 a short aliphatic chain. In short, position N1 would appear to be the preferred site for functionalization for subsequent conjugation, since a wide variety of substitution patterns are tolerated at this position. Our initial concept was to synthesize halogenated 1‐benzyl‐2‐butyl‐1*H*‐imidazo[4,5‐*c*]quinolin‐4‐amines, which then act as substrates for cross‐coupling reactions to install various keto‐functionalities. To ensure rapid pH‐driven dissociation, an aliphatic ketone was introduced. Considering the circumstances mentioned above, a synthesis route was designed starting with 4‐chloro‐3‐nitroquinoline, a common precursor for fused quinoline‐type heterocyclic frameworks (Scheme 1).^20^ A functionalized benzylic group can be installed at this stage by substitution of the C4‐chlorine with a benzylamine. Treatment of 4‐chloro‐3‐nitroquinoline with *2*‐bromo benzylamine using triethylamine in THF solution gave quinolin‐4‐amine **1** in excellent yield. By combining and adjusting two published one‐pot procedures for the formation of benzimidazoles starting from *ortho*‐nitroaniline,^21^ 1‐benzyl‐2‐butyl‐1*H*‐imidazo[4,5‐*c*]quinoline **2** could be synthesized in 83 % yield. This transformation consisted of the reduction of the nitro group with iron powder aided by ammonium chloride in *iso*‐butanol combined with the annulation of a 1*H*‐2‐butyl‐imidazole motif employing valeraldehyde. The established route to carry out amino‐functionalizations with quinoline‐derivatives is to firstly subject compounds such as **3** to N‐oxidization (compare Scheme 1, **4**), before conversion with nitrogen‐bearing reagents. Aliphatic ketones could be successfully installed in a single step via a Heck‐type reaction using an allyl alcohol along with triethylamine,^22^ to give a 3‐oxo‐butyl‐chain attached to the benzyl‐group. By subjecting various halobenzyl‐imidazoquinoline derivatives to this palladium acetate‐catalyzed coupling, suitable conversions were observed. By far the highest‐yielding reaction was shown to be preparation of **3** from **2** with but‐3‐en‐2‐ol (81 %, Scheme 1). The *N*5‐oxide **4** could be accessed in very good yield by employing *meta*‐chloroperoxybenzoic acid as an oxidizing agent, without the requirement for hitherto unsuccessful ketal protection of the ketones. These formations proceeded slowly, taking 60 h. The same was true for the final preparation of C4‐amines, where the reaction conditions also involved refluxing in dichloromethane for two to three days. For this purpose, benzoyl isocyanate was added as the nitrogen source. The concluding step consisted of refluxing the intermediate with sodium methoxide in methanolic solution to release the primary amine. The synthesis of **5** resulted in good yield (53 %). In order to assess the effect of chemical modification on the activity of the novel TLR agonist, a series of tests were carried out in living zebrafish larvae as shown in Figure 1. Stimulation with **5** resulted in NF‐kB signaling activation in zebrafish macrophages visualized in Tg(mpeg1:mCherry)^gl23 [23]^/Tg(6×Hsa. NFKB: EGFP)^nc1 [24]^ double transgenic animals, which have macrophages demarcated by mCherry expression and report NF‐κB activity by EGFP expression (Figure 1 A).

**Scheme 1:**
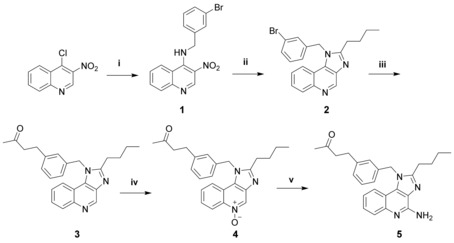
Reagents and conditions: (i) 2‐bromobenzylamine, NEt_3_, THF, RT; (ii) iron powder, NH_4_Cl, valeraldehyde, 2‐BuOH, air, reflux; (iii) but‐3‐en‐2‐ol, Pd(OAc)_2_, NEt_3_, DMA, 130 °C; (iv) mCPBA, CH_2_Cl_2_, reflux; (v) I.) benzoyl isocyanate, CH_2_Cl_2_, reflux; II.) NaOMe, MeOH, reflux.

**Figure 1:**
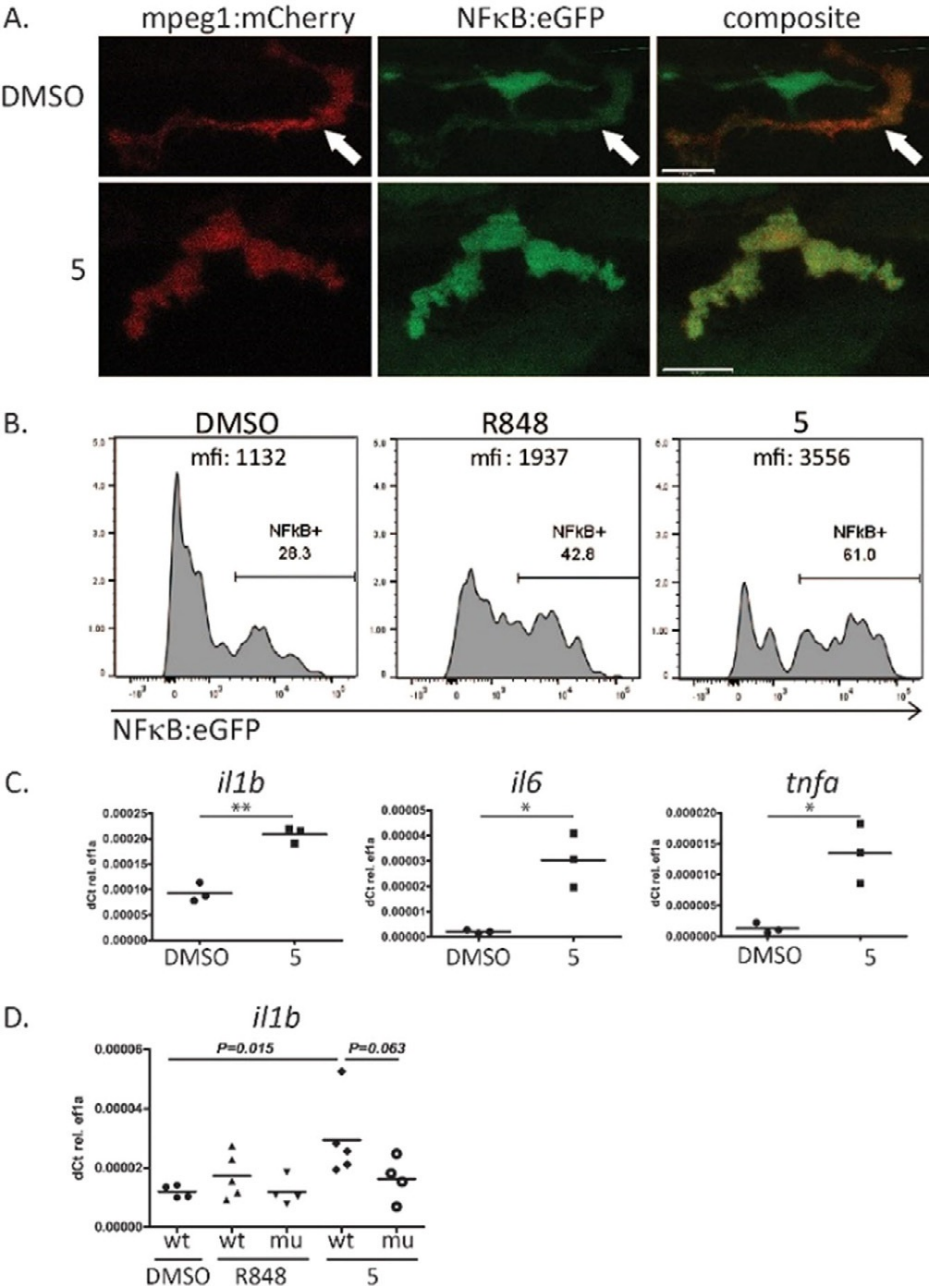
In vivo studies of the immune‐ activating functions of **5**. (A) and (B) NFκB:eGFP induction by **5** in mpeg:mCherry positive macrophages in live zebrafish larvae. Zebrafish larvae (4 dpf) were stimulated with DMSO (control), R848 (positive control) or **5** for 6 h. A) Representative confocal images of macrophages in live NFκB:eGFP/mpeg:mCherry transgenic larvae. Upper panel: DMSO control (arrow demarcates macrophage). Lower panel: **5** (B) Flow cytometry analysis of NF‐κB induction in single‐cell suspensions of zebrafish larvae. Cells were pre‐gated on mpeg:mcherry macrophages and analysed for NFκB:eGFP expression. (C) Quantitative real‐ time PCR for RNA expression of the inflammatory cytokines il1b, il6, tnfa in wildtype zebrafish larvae after 6 h activation with **5**. DMSO was used as negative control. (*n*=3) *P<0.05; ** P<0.01 (D) Il1b RNA expression in MyD88 mutant and wildtype zebrafish larvae after 6 h of stimulation with TLR ligands R848 and **5**. DMSO was used as negative control. (*n*=4–5) Scale bars in A are 10 μm.

**5** showed stronger induction of NF‐κB than the known TLR7/8 agonist R848 as quantified by flow cytometry (Figure 1 B). QPCR further confirmed induction of inflammatory cytokines IL1b, IL6 and Tnf‐α upon **5** stimulation for 6 h (Figure 1 C). In addition, IL1b induction upon stimulation with 5 was reduced in MyD88 mutant zebrafish, indicating a MyD88 dependent signal pathway as expected for specific TLR7/8 activation (Figure 1 D).^25^ In the next step, water soluble polyphosphazenes decorated with hydrazide linkers (43 wt %) were prepared as previously reported (see ref. 26 and Supporting Information Figure SI‐2). The novel TLR agonist **5** could be successfully bound to the polymer due to hydrazone formation with the carbonyl groups (Scheme 2). After purification by dialysis, UV/Vis, GPC and DLS measurements were used to prove covalent drug loading. The percentage of loading was calculated using the UV/Vis absorbance at 324 nm (Figure SI‐3). Conjugation between 3 and 6 weight percent of the macromolecular conjugate was achieved representing a loading yield of between 10 and 25 percent of the linkers (Figure SI‐3).

**Scheme 2:**
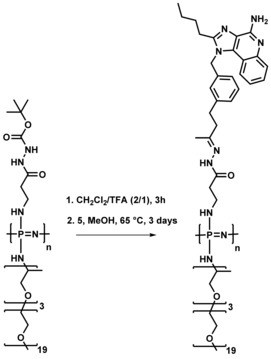
Chemical conjugation of TLR agonist **5** to the poly(organo)‐phosphazene (simplified structure) via a hydrazone linkage.

The GPC curves of the polymer conjugates after drug loading, measured in DMF, are shifted to lower retention volumes (lower hydrodynamic volume), presumably due to the hydrophobicity of the conjugated drugs, while still possessing a relatively narrow Đ of 1.3 (Figure SI‐4). The hydrodynamic volume is a crucial parameter in terms of bio‐distribution and cell uptake. Dynamic light scattering studies showed that whilst the hydrophilic polymers show hydrodynamic diameters in the 10 nm range, they form supramolecular associates in the 100 nm range upon conjugation of the hydrophobic drug as a result of hydrophobic interactions (Figure 2). This phenomenon which has been previously reported with similar phosphazene based systems^27^ is thought to be assisted by the high backbone flexibility relative to carbon based polymers. Furthermore, in a work with different polymer‐TLR7/8 agonist conjugates, Lynn et al. recently showed that increased densities of Toll‐like receptor agonists arrayed on polymer carriers is associated with particle formation and enhanced cytokine production.^28^ Double internalization of the agonist, via its chemical binding location close to the backbone of the brush type polymers is further pronounced by the agglomeration of the hydrophobic units in aqueous solutions, thus offering the potential for a so‐called ‘stealth‐effect’,^29^ reducing the probability of agonist presentation prior to intracellular release. The release of **5** from the conjugates was analyzed by HPLC. Within a period of 24 h at 37 °C, 100 % release from the polymer–drug conjugates could be observed for the samples exposed to pH 5 and only 50 % for the conjugates stored at pH 7.4 (Figure 2 b).

**Figure 2:**
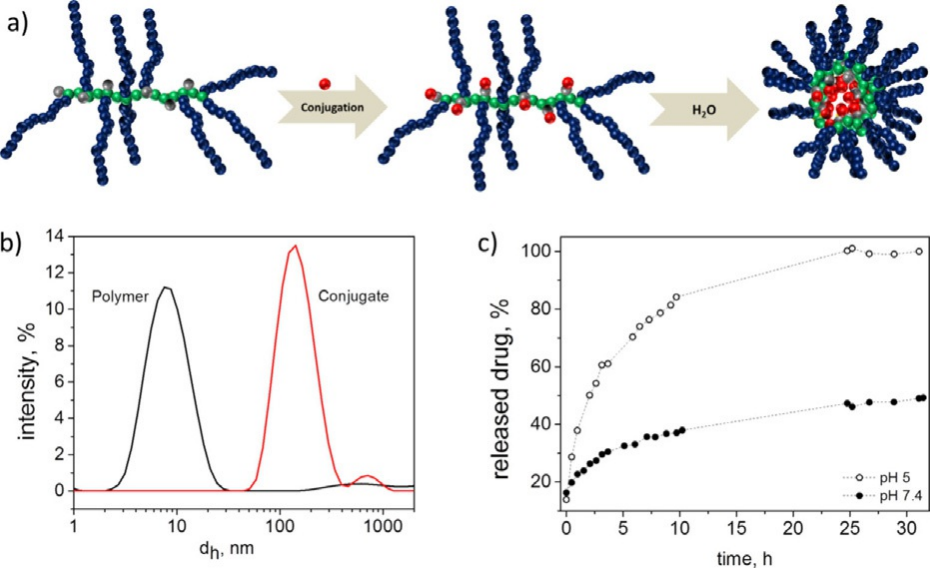
a) Schematic representation of the intra‐ and intermolecular agglomeration and self‐assembly of the polymers upon conjugation of the hydrophobic drug in aqueous solvents. b) Molecular size distribution by intensity as detected by dynamic light scattering for polymers 2–5 in phosphate buffer at pH 7.4 (polymer concentration 1 mg mL^−1^, d_h_—hydrodynamic diameter). c) Release of **5** from the conjugate at 37 °C in acidic environment (acetate buffer, pH 5), and a neutral solution (pH 7.4, tris buffer). The amount of the released drug was estimated using a calibration curve for the free drug.

The release rate is comparable to published data using similar hydrazide based polymer systems.^26^, ^30^ At pH 5, approximately 50 % release of compound **5** was observed after 2.5 hours and full release was observed after 30 hours, whereas at pH 7.4 only approx. 50 % release of **5** was observed during the whole period measured. As complete clearance of the macromolecular carrier is a stringent requirement for subsequent in vivo applications, the degradation profiles of the polymers were also investigated. The degradation studies of the conjugates at 37 °C, pH 5 and 7 measured by size exclusion chromatography, showed that the polymers are stable over a short period of time in an aqueous environment but degrade significantly to small molecules under these simulated physiological conditions within 10 weeks (SI‐5). These results are comparable to previous degradation studies of amino substituted poly(organo)phosphazenes but could be easily accelerated or decelerated as and when required.^11^ Compound **5**, upon release from polymer‐ImQ conjugate is expected to stimulate T cells through internalization and activation of APCs such as DCs. Thus, in order to analyse the immunological activity of polymeric prodrugs we started investigating cellular uptake and localization of ImQ‐conjugated within murine DCs. Compound 5‐conjugated ImQ polymer accumulated in generated DCs and co‐localized with intracellular endosomal and lysosomal vesicles (Figure 3 A–D). Overall, a total of 87 % of all DCs internalized ImQ‐based macromolecular prodrug.

**Figure 3:**
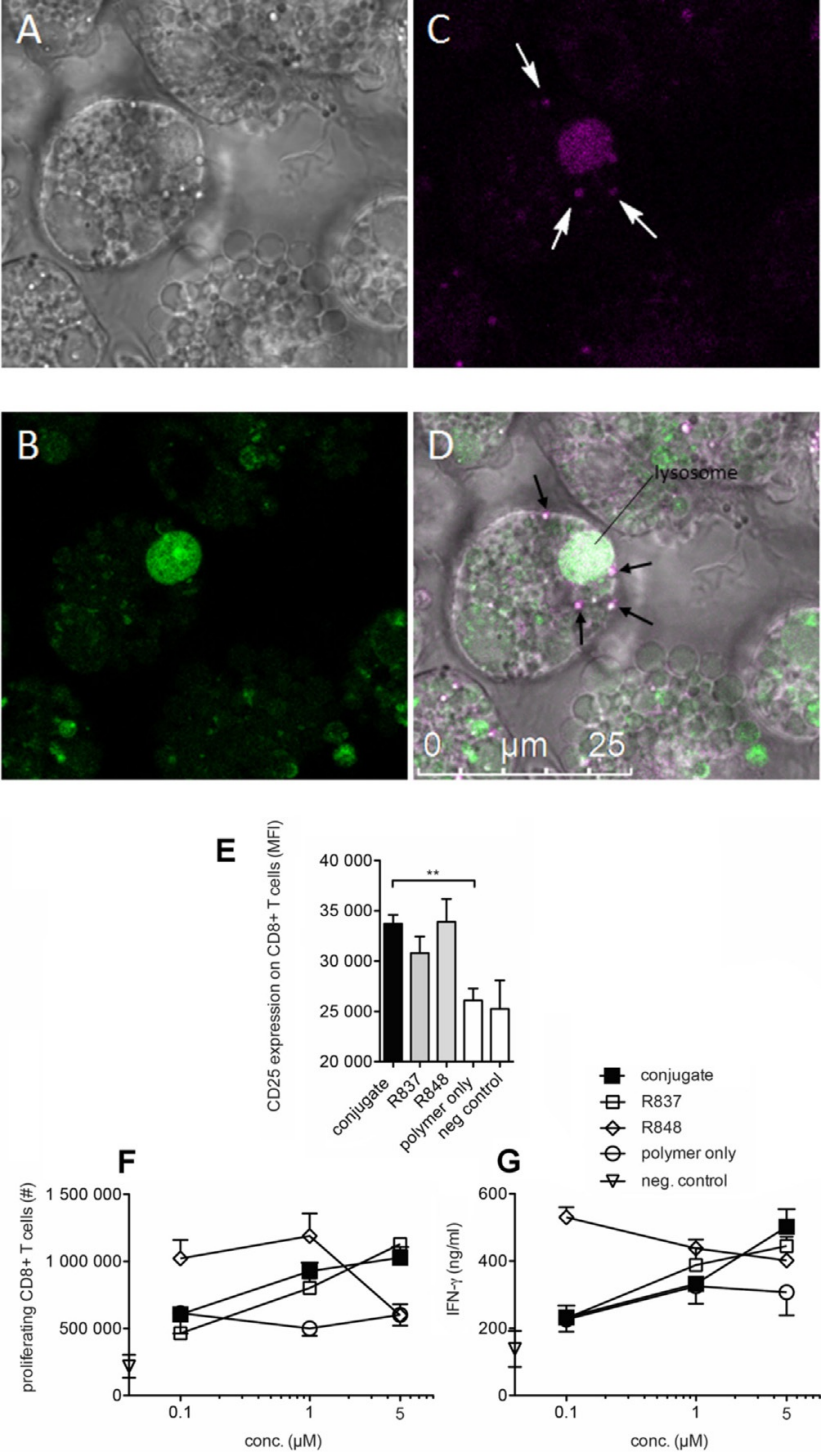
A–D: Confocal microscopy images of dendritic cells incubated with polymer‐ ImQ conjugates. Murine BMDCs (A) were stained with LysoTracker Green (504 nm/511 nm) for 1 hour (B) and with polymer‐ImQ (390 nm/415 nm). LysoTracker Green accumulates in cellular compartments with low internal pH and stains lysosomes (green, B). Polymer‐ImQ accumulates in lysosomes and in endosomes (C and D, purple regions indicated by arrows). E–F) Conjugates activate T cells and IFN‐γ release. Splenocytes were isolated from transgenic OT‐I mice, labeled with CFSE and stimulated for 4 days with R837, R848 or conjugate, together with the ovalbumin derived SIINFEKL‐peptide, which stimulates ovalbumin specific CD8+T cells from OT‐I mice. On day 4, CD8+T cells were analyzed for CD25 expression and CFSE diluted cells, considered as proliferating T cells by flow cytometry. E) CD25 expression depicted as mean fluorescence intensity (MFI) is shown for splenocytes cultures containing 1 μm conjugate. F) Absolute cell numbers of proliferating CD8+T cells per splenocyte culture were calculated in CFSE diluted dividing T cells using a dosage ranging from 0.1 to 5 μm. G) Supernatants from splenocyte cultures were analyzed for IFN‐γ secretion. Mean ± standard deviation from triplicate analysis is shown for CD25 expression, *) p<0.05, **) p<0.01. Polymer alone and SIINFEKL peptide alone dissolved in DMSO served as negative controls.

The capacity of ImQ‐conjugate activated DCs to stimulate T cells via DCs was further analysed in T cell/DC co‐cultures.

We used splenocytes from OT‐I mice, which among other immune cells comprise APCs and predominantly CD8+T cells. Such T cells entirely express a transgenic T cell receptor, which is specific for a short ovalbumin related peptide sequence (SIINFEKL) primarily presented by APCs. Thus strong APC‐mediated T cell responses can be raised in an OT‐I splenocyte culture upon TLR7/8 ligation together with SIINFEKL‐peptide. In order to investigate the effect on T cells, we measured the degree of T cell activation, as well as the secretion of IFN‐γ, a cytokine that is associated with activation of CD8+ effector T cells (Figure 3 E–F). The activation marker CD25 was significantly increased on CD8+T cells in stimulation cultures using conjugates, R837 or R848 compared to the pristine polymer alone. The dose response curve using the conjugate was comparable to the course of proliferation induced by R837, between 0.1 and 5 μm. The amount of IFN‐γ released into the culture was between 200 and 600 ng mL^−1^ after the stimulation with 0.1 to 5 μm using conjugate, R837 or R848 compared to 200 ng mL^−1^ for the polymer backbone or SIINFEKL‐peptide alone. The conjugates induced the highest IFN‐γ release at 5 μm, which strongly declined with decreasing conjugate concentrations in the splenocytes cultures down to levels of the negative controls as shown by polymer or SIINFEKL peptide alone. Based on our observation the conjugate strongly stimulates murine CD8+T cells presumably by DC‐mediated T cell priming involving TLR7/8 signaling in DCs.

In conclusion, the chemical synthesis and biological activity of a novel functionalized imidazoquinoline (ImQ) derivative bearing a 3‐(3‐oxobutyl)‐benzyl moiety at the N1‐position of the ImQ was presented. The keto‐group was introduced to provide a site for reversible binding to hydrazide groups attached to modified, water soluble and biodegradable polyphosphazenes. The resulting hydrazone functionality served as an acid labile linkage between the polymeric carrier system and the immune response modifier. Compound 5‐conjugated ImQ polymer accumulated in generated DCs and co‐localized with endosomal and intracellular lysosomal vesicles. The capacity of ImQ‐conjugate activated DCs to stimulate T cells via DCs was further analysed in T cell/DC co‐cultures.

In splenocytes from OT‐I mice stimulated with polymer‐ImQ conjugates in the presence of SIINFEKL‐antigen, CD8+T cells proliferated and strongly released IFN‐γ into the supernatant when compared to known immune response modifiers resiquimod (R848) and imiquimod (R837), used as positive controls. In addition, CD8+T cells showed high expression of the IL‐2 receptor (CD25), considered as an activation marker for T cells after polymer‐ImQ conjugate stimulation. Ongoing in vivo studies on murine tumor models are in progress. Our efforts will focus to target the tumor microenvironment of tumors to induce local inflammation, which might lead to tumor rejecting immune reactions.

## Experimental Section

Full experimental details, including details on the materials used, synthetic procedures and structural characterisation can be found in the Supporting Information.

### Zebrafish husbandry

Zebrafish (*Danio rerio*) larvae were bred in our facility under institutional and personal animal research licenses (GZ: 565304/2014/6; GZ: 534619/2014/4) according to the guidelines of the local authorities. Zebrafish were raised under standard conditions at a temperature of 28 °C in a research fish facility (Tecniplast, Italy) with circulating and constantly filtered water at pH 7.5 and a conductivity of around 550 μS. Eggs were bleached with 0.005 % sodium hypochlorite at 1 dpf and kept in egg medium E3 with 20 mg/l phenylthiourea (PTU) (Sigma–Aldrich, St.Louis, MO). Wildtype fish were SAT [1]. The following previously described transgenic lines were used: *Tg(mpeg1:mCherry)* ^*gl23*^ [2], *Tg(6xHsa.NFKB:EGFP)* ^*nc1*^ [3], *myd88* ^*hu3568/hu3568*^ [4]. *C57BL/6‐ Tg(TcraTcrb)1100Mjb/J (OT‐I)* mice were purchased from Charles River Laboratories (Sulzfeld, Germany). All murine animal experiments were performed in accordance with the institutional guidelines and approved by the Animal Care and Use Committee of the Medical University of Vienna (GZ: 856861/2013/16).

## Conflict of interest

The authors declare no conflict of interest.

## Supporting information

As a service to our authors and readers, this journal provides supporting information supplied by the authors. Such materials are peer reviewed and may be re‐organized for online delivery, but are not copy‐edited or typeset. Technical support issues arising from supporting information (other than missing files) should be addressed to the authors.

Supplementary
