# Supplementary material for: A pH‐sensitive Macromolecular Prodrug as TLR7/8 Targeting Immune Response Modifier
Source: Chemistry. 2017 Sep 4;23(70):17721–6. doi: 10.1002/chem.201702942 (PMC5763314; doi:10.1002/chem.201702942)
Supplement: Supplementary file 1 — Supplementary [file CHEM-23-17721-s001.pdf]

CHEMISTRY
A European Journal

Supporting Information

A pH-sensitive Macromolecular Prodrug as TLR7/8 Targeting Immune Response Modifier

Stefan Aichhorn+,[a] Anne Linhardt+,[b] Angela Halfmann,[c] Markus Nadlinger,[a] Stefanie Kirchberger,[c] Manuela Stadler,[c] Barbara Dillinger,[c] Martin Distel,*[c] Alexander Dohnal,*[c] Ian Teasdale,*[b] and Wolfgang Schöfberger*[a]

chem_201702942_sm_miscellaneous_information.pdf

Experimental

Materials and Methods

All solvents were dried using standard laboratory procedures. Synthetic of polymers were carried out either in a glove box (MBRAUN) under argon or under nitrogen using standard Schlenk line techniques. The polyetheramine copolymer (PEO-PPO-NH_2) with an ethylene oxide / propylene oxide ratio of 19/3 and a M_n of 1000 g mol^{-1}, sold under the trade name Jeffamine M-1000, was donated by Huntsman Performance Products and used as received. PCl_5 was purified by sublimation and stored under argon. Triethylamine was distilled and dried over molecular sieves prior to use. All other chemicals were purchased from Sigma Aldrich, Acros Chemicals, or Alfa Aesar, and used without further purification. All glassware was dried in an oven overnight prior to use.

^{1}H and ^{13}C NMR spectra were recorded on a Bruker Ascend 700 MHz Avance III NMR spectrometer equipped with a cryoprobe, DRX 500 MHz NMR spectrometer equipped with a cryoprobe (TXI), or on a Bruker Avance 300 MHz spectrometer. The chemical shifts are referenced to the residual nondeuterated solvent. ^{31}P NMR spectra were recorded on a DRX 500 MHz NMR spectrometer at 202.4 MHz. High resolution mass spectra were collected on a Thermo Fisher Scientific LTQ Orbitrap XL with an Ion Max API Source. UV-Vis spectra were carried out on a Perkin Elmer Lambda 25 UV/VIS spectrophotometer. Size-exclusion chromatography was carried out on a Viscotek GPCMax High Temperature SEC (HT-SEC) System equipped with PFG columns from PSS, (Mainz, Germany) 300 x 8 mm^2, 5 µm particle sizes, a Model 350 High Temperature Triple Detector Array (HT-TDA) featuring a Differential Refractive Index (RI) Detector, four-capillary Differential Viscometer Detector and patent-pending Low Angle Light Scattering (LALS) Detector, Vortex Autopreparation/Autosampler Module for automated sample preparation and delivery, Model 1122 Isocratic Pump for reproducible flow rates with an absence of pump pulsation, Model 7510 Degasser for elimination of air and other solvent gases and OmniSEC software for complete system control, data acquisition and processing. The samples were eluted with DMF containing 10 mM LiBr at a flow rate of 0.75 mL min^{-1} at 60°C. The molecular weights were estimated using a conventional calibration of the refractive index detector versus linear polystyrene standards (S-L-10). A 1290 Infinity UHPLC from Agilent Technologies (Agilent, Vienna, Austria) equipped with a reversed-phase C18 silica-based chromatographic column (Rapid Resolution HD Eclipse Plus C18; 2.1 mm x 50 mm, particle size 1.8 µm) was used for kinetic studies of the drug release. The samples were eluted at a flow rate of 0.3 mL min^{-1} at room temperature with a mobile phase composition of 40 % acetonitrile in water (v/v) containing 0.1% formic acid (v/v)

in isocratic mode. UV detection was carried out at 260 nm. The amount of the released drug was estimated using a calibration curve for the free drug. A Malvern ZetaSizer Nano-ZS analyzer (Malvern Instruments, Malvern, UK) was used for dynamic light scattering (DLS) investigations with the 4 mW HeNe laser set at λ = 633 nm and the detector angle at 173° for backscattering measurements. The measurements were carried out in phosphate buffer (1 mg/mL) and all samples were filtered through a Millipore Millex-GV (Billerica, Massachusetts, USA) 0.22 µm PVDF filter and measured in a disposable polystyrene ultra-micro cuvette at 25 °C.

Zebrafish husbandry

Zebrafish (*Danio rerio*) larvae were bred in our facility under institutional and personal animal research licenses (GZ: 565304/2014/6; GZ: 534619/2014/4) according to the guidelines of the local authorities. Zebrafish were raised under standard conditions at a temperature of 28 °C in a research fish facility (Tecniplast, Italy) with circulating and constantly filtered water at pH 7.5 and a conductivity of around 550 µS. Eggs were bleached with 0.005 % sodium hypochlorite at 1 dpf and kept in egg medium E3 with 20 mg/l phenylthiourea (PTU) (Sigma-Aldrich, St.Louis, MO). Wildtype fish were SAT [1]. The following previously described transgenic lines were used: $Tg(mpeg1{:}mCherry)^{gl23}$ [2], $Tg(6xHsa.NFKB{:}EGFP)^{nc1}$ [3], $myd88^{hu3568/hu3568}$ [4].

Confocal microscopy

mpeg1:mCherry/ NFκB:eGFP double- positive larvae (4 dpf) were treated with 5 (5 µM) or DMSO in E3/PTU for 6 h. Larvae were anaesthetised with 0.02 % tricaine and embedded in 1.2 % low-melting agarose (Sigma-Aldrich) as described previously [5]. Confocal images were acquired on a TCS SP8 WLL microscope (Leica, Wetzlar, Germany). Image analysis was performed using the Leica LAS software.

Flow cytometry

mpeg1:mCherry/ NFκB:eGFP double- positive larvae (4 dpf) were stimulated with R848 (5 µM), 5 (5 µM) or DMSO in E3/PTU for 6 h. Larvae were anaesthetised using tricaine and immersed in 10 mM DTT (Sigma-Aldrich) in E3 for 30 min to remove the mucus. Single cell suspensions from larvae were prepared using Liberase Blendzyme TM at 1.1 U/ml and Dnase I at 40 µg/ml (both Sigma-Aldrich) in HBSS (Life technologies, Carlsbad, CA) under constant

shaking at 37°C. Cells were washed in PBS/BSA and analysed on an LSRFortessa cytometer (Becton-Dickinson, Franklin Lakes, NJ).

Quantitative real-time PCR

Wildtype SAT zebrafish larvae (3 dpf) or MyD88$^{mu/+}$ offspring larvae were stimulated for 6 h as described above. For analyses of cytokines (*il1b, il6, tnfa*) in wildtype larvae 10 larvae/ replicate were used. For analysis of *il1b* in MyD88$^{mu/+}$ offspring, single larvae were used to allow individual genotyping for MyD88 status. Larvae were anaesthetised using tricaine, frozen in liquid nitrogen and the tissue disrupted in RLT buffer supplemented with 2-mercaptoethanol using a pestle. RNA was isolated using the RNeasy Mini kit (Qiagen, Germany). RNA was transcribed with a High Capacity cDNA transcription kit (Applied Biosystems, Foster City, CA). For the MyD88 experiment single larvae were genotyped from cDNA by performing a standard PCR (primers: MyD88 fw 5´- TTGACGGACT GGGAAACTCG -3´; MyD88 rev 5´-AGGTGTCAGTCCCTGTGGAT-3´) and by sequencing of the product for the MyD88 mutation described in **van den Vaart, DMM, 2013**. qPCR was performed with a Maxima SYBR green mix on a 7500 Fast real-time PCR machine (both Applied Biosystems). Following primers were used for qPCR: il1b fw 5´- AAAGTGCGCTTCAGCATGTC-3´; il1b rev 5´-ACCCGCTGATCTCCTTGAGT-3´; il6 fw 5´- GTCCCCGTGTTCAGCAGTAT-3´; il6 rev 5´- CGCGTTAGACATCTTTCCGTG-3´; tnfa fw 5´- TTCACGCTCCATAAGACCCA-3´; tnfa rev 5´- CCGTAGGATTCAGAAAAGC G-3´.

Synthesis of imidazoquinoline derivatives

Our initial concept was to synthesize halogenated 1-benzyl-2-butyl-1*H*-imidazo[4,5-*c*]quinolin-4-amines, which then act as substrates for cross-coupling reactions to install various keto-functionalities. To ensure rapid pH-driven dissociation, an aliphatic ketone was introduced. Considering the circumstances mentioned above, a synthesis route was designed starting with 4-chloro-3-nitroquinoline, a common precursor for fused quinoline-type heterocyclic frameworks (Scheme 1) [6]. A functionalized benzylic group can be installed at this stage by substitution of the C4-chlorine with a benzylamine. Treatment of 4-chloro-3-nitroquinoline with *2*-bromo benzylamine using triethylamine in THF solution gave quinolin-4-amine **1** in excellent yield. By combining and adjusting two published one-pot procedures for the formation of benzimidazoles starting from *ortho*-nitroaniline [7], 1-benzyl-2-butyl-1*H*-imidazo[4,5-*c*]quinoline **2** could be synthesized in 83 % yield. This transformation consisted of the reduction of the nitro group with iron powder aided by ammonium chloride in *iso*-butanol combined with the annulation of a 1*H*-2-butyl-imidazole motif employing valeraldehyde. The

established route to carry out amino-functionalizations with quinoline-derivatives is to firstly subject compounds such as **3** to *N*-oxidization (compare Scheme 1, **4**), before conversion with nitrogen-bearing reagents. Aliphatic ketones could be successfully installed in a single step via a Heck-type reaction using an allyl alcohol along with trimethylamine [8], to give a 3-oxo-butyl-chain attached to the benzyl-group. By subjecting various halobenzyl-imidazoquinoline derivatives to this palladium acetate-catalyzed coupling, suitable conversions were observed. By far the highest-yielding reaction was shown to be preparation of **3** from **2** with but-3-en-2-ol (81 %, Scheme 1). The *N*5-oxide **4** could be accessed in very good yield by employing *meta*-chloroperoxybenzoic acid as an oxidizing agent, without the requirement for hitherto unsuccessful ketal protection of the ketones. These formations proceeded slowly, taking 60 h. The same was true for the final preparation of C4-amines, where the reaction conditions also involved refluxing in dichloromethane for two to three days. For this purpose, benzoyl isocyanate was added as the nitrogen source. The concluding step consisted of refluxing the intermediate with sodium methoxide in methanolic solution to release the primary amine. The synthesis of **5** resulted in good yield (53 %).

Synthesis of **1** (*N*-(3-bromobenzyl)-3-nitroquinolin-4-amine):

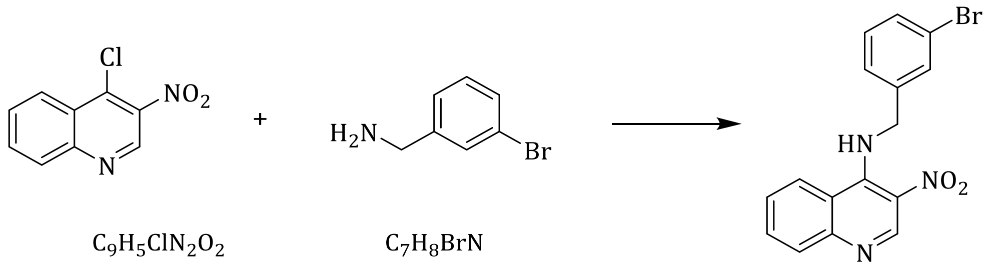

1

$C_{16}H_{12}BrN_3O_2$

To a solution of 1-chloro-2-nitroquinoline (1.858 g, 8.91 mmol, 1 equiv.) in 50 ml dichloromethane were added triethylamine (2.0 ml, 14.0 mmol, 1.6 equiv.) and 3-bromobenzylamine (1.988 g, 10.7 mmol, 1.2 equiv.). Upon refluxing under N_2 atmosphere for 24 h, yellow precipitate formed. The mixture was concentrated under reduced pressure and the residue was taken up in pentanes. The mixture was stirred at 35°C for 30 min and the resulting yellow suspension was cooled to ambient temperature and filtered. The residue was washed with pentanes and dried *in vacuo* to afford the title compound as bright yellow solid (3.087 g, 8.64 mmol, 97%). M.p. 165–168°C; ^{1}H NMR (300 MHz, $CDCl_3$, 298 K): δ = 5.08 (d, J = 6.0 Hz, 2H), 7.31–7.39 (m, 2H), 7.45 (t, J = 7.7 Hz, 1H), 7.53 (d, J = 7.4 Hz, 1H), 7.59 (s, 1H), 7.79 (t, J = 7.6 Hz, 1H), 8.04 (d, J = 8.3 Hz, 1H), 8.18 (d, J = 8.5 Hz, 1H), 9.42 (s, 1H), 9.80 (br s, 1H) ppm; ^{13}C NMR (126 MHz, $CDCl_3$, 298 K): δ = 52.4, 119.2, 123.6, 125.7, 125.9,

126.7, 126.8, 130.4, 130.8, 131.1, 131.8, 133.0, 139.3, 147.4, 150.8, 151.1 ppm; HRMS (ESI) *m/z* calcd for $C_{16}H_{12}BrN_3O_2$: 358.0186 $[M+H]^+$, found: 358.0185.

Synthesis of **2** (2-butyl-1-(3-bromobenzyl)-1*H*-imidazo[4,5-*c*]quinoline)**:**

1

$C_{16}H_{12}BrN_3O_2$

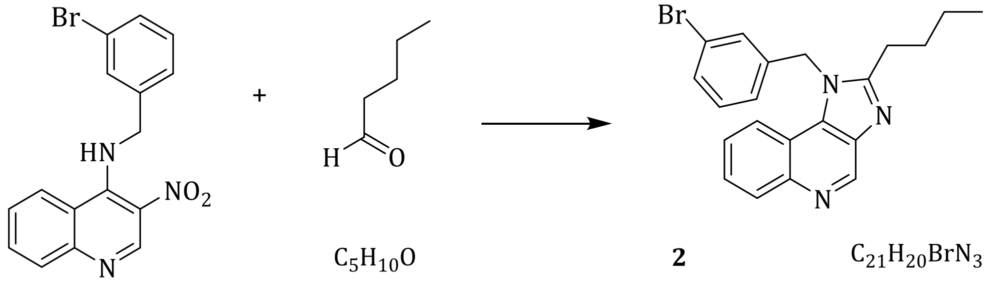

$C_5H_{10}O$

2 $C_{21}H_{20}BrN_3$

A suspension of **1** (1.278 g, 3.57 mmol, 1 equiv.), iron powder (1.989 g, 35.7 mmol, 10 equiv.), NH_4Cl (1.910 g, 35.7 mmol, 10 equiv.), and *n*-pentanal (1.72 ml, 17.9 mmol, 5.0 equiv.) in 40 ml *iso*-butanol was refluxed under air for 2 h. After cooling to ambient temperature, the mixture was treated with 1 M NaOH and EtOAc. Phases were separated and the aqueous layer was extracted with EtOAc twice. The combined organic layers were washed with 1 M NaOH twice, dried over anhydrous Na_2SO_4 and concentrated. Heptanes were slowly added at 60°C. Upon cooling at –10°C overnight, the generated solid was filtrated, washed with cold heptanes and dried *in vacuo* to afford the title compound as yellow solid (1.177 g, 2.96 mmol, 83%).

M.p. 146–148°C; ^1H NMR (300 MHz, $CDCl_3$, 298 K): δ = 0.95 (t, *J* = 7.3 Hz, 3H), 1.47 (sext, *J* = 7.5 Hz, 2H), 1.89 (quint, *J* = 7.7 Hz, 2H), 2.92 (t, *J* = 7.8 Hz, 2H), 5.78 (s, 2H), 6.88 (d, *J* = 7.7 Hz, 1H), 7.18 (t, *J* = 7.9 Hz, 1H), 7.29 (s, 1H), 7.41–7.49 (m, 2H), 7.62 (ddd, *J* = 1.1 Hz, 7.7 Hz, 1H), 7.87 (d, *J* = 8.4 Hz, 1H), 8.27 (d, *J* = 8.4 Hz, 1H), 9.36 (s, 1H) ppm; ^{13}C NMR (176 MHz, $CDCl_3$, 298 K): δ = 13.9, 22.7, 27.3, 29.7, 48.5, 117.6, 119.7, 123.7, 124.1, 126.7, 127.1, 128.8, 131.1 (2C), 131.6, 134.1, 136.7, 137.7, 145.0, 145.2, 155.8 ppm; HRMS (ESI): *m/z* calcd for $C_{21}H_{20}BrN_3$: 394.0913 $[M+H]^+$; found: 394.0911.

Synthesis of **3** (4-(3-((2-butyl-1*H*-imidazo[4,5-*c*]quinolin-1-yl)methyl)phenyl)butan-2-one):

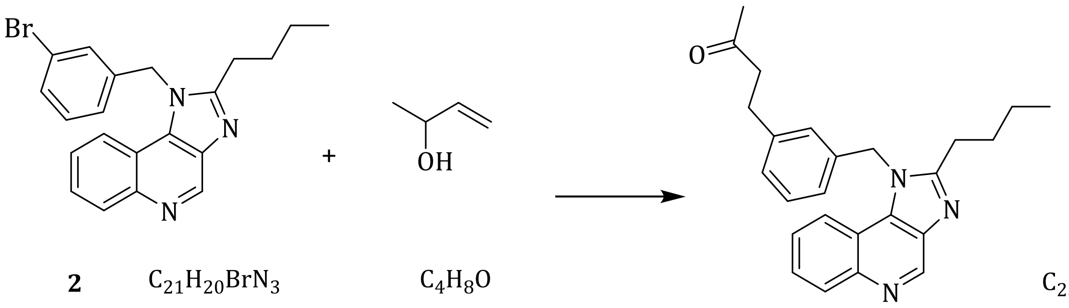

2 $C_{21}H_{20}BrN_3$ C_4H_8O **3** $C_{25}H_{27}N_3O$

To a solution of **2** (368 mg, 0.90 mmol, 1 equiv.) in 6 ml *N*,*N*-dimethylacetamide, but-3-en-2-ol (113 µL, 1.26 mmol, 1.4 equiv.), triethylamine (177 µL, 1.26 mmol, 1.4 equiv.) and palladium(II) acetate (0.044 mmol, 10 mg, 0.05 equiv.) were added. The mixture was stirred at 130°C under N_2 atmosphere for 26 h. After cooling to ambient temperature, 0.5 M $NaHCO_3$ was added and the resulting aqueous layer was extracted with EtOAc three times. The combined organic layers were washed with brine, dried over anhydrous Na_2SO_4 and concentrated under reduced pressure. The residue was purified by column chromatography (silica gel, DCM/MeOH = 11 / 1) to afford the title compound as colorless oil (284 mg, 0.73 mmol, 81%).

^{1}H NMR (300 MHz, $CDCl_3$, 298 K): δ = 0.92 (t, *J* = 7.3 Hz, 3H), 1.44 (sext, *J* = 7.5 Hz, 2H), 1.86 (quint, *J* = 7.7 Hz, 2H), 2.01 (s, 3H), 2.60 (t, *J* = 7.4 Hz, 2H), 2.79 (t, *J* = 7.4 Hz, 2H), 2.90 (t, *J* = 7.8 Hz, 2H), 5.72 (s, 2H), 6.81 (d, *J* = 7.7 Hz, 1H), 6.86 (s, 1H), 7.09 (d, *J* = 7.6 Hz, 1H), 7.20 (t, *J* = 7.6 Hz, 1H), 7.39 (ddd, *J* = 1.2 Hz, 7.6 Hz, 1H), 7.57 (ddd, *J* = 1.2 Hz, 7.7 Hz, 1H), 7.87 (d, *J* = 8.4 Hz, 1H), 8.22 (d, *J* = 8.4 Hz, 1H), 9.34 (s, 1H) ppm; ^{13}C NMR (125 MHz, $CDCl_3$, 298 K): δ = 13.9, 22.6, 27.3, 29.5, 29.7, 30.1, 44.8, 49.0, 117.7, 120.0, 123.4, 125.6, 126.5, 126.9, 128.3, 129.6, 130.6, 134.3, 135.4, 136.5, 142.5, 144.6, 144.8, 156.1, 207.5 ppm; HRMS (ESI): *m/z* calcd for $C_{25}H_{27}N_3O$: 386.2227 $[M+H]^+$; found: 386.2226.

Synthesis of **4** (2-butyl-1-(3-(3-oxobutyl)benzyl)-1*H*-imidazo[4,5-*c*]quinoline 5-oxide):

3

$C_{25}H_{27}N_3O$

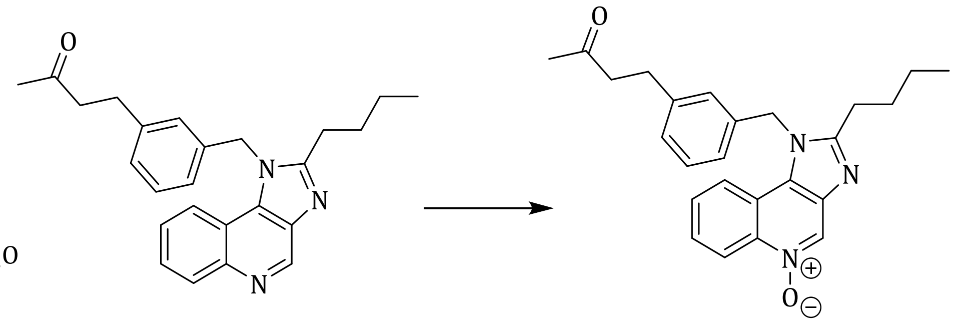

4

$C_{25}H_{27}N_3O_2$

To a solution of **3** (286 mg, 0.74 mmol, 1 equiv.) in 35 ml dichloromethane, 3-chloroperoxybenzoic acid (1.85 mmol, 415 mg, 77%, 2.5 equiv.) was added. The mixture was heated at reflux under N_2 atmosphere for 63 h. Then, the solvent was removed and the residue dissolved in EtOAc, treated with 1 M $NaHCO_3$ solution and thoroughly stirred at room temperature for 2 h. Phases were separated and the aqueous layer extracted with EtOAc. The combined organic layers were dried over anhydrous Na_2SO_4, evaporated under reduced pressure and gave the title compound as pale yellow oil (226 mg, 0.56 mmol, 76%).

^{1}H NMR (300 MHz, $CDCl_3$, 298 K): δ = 0.93 (t, J = 7.4 Hz, 3H), 1.44 (sext, J = 7.5 Hz, 2H), 1.84 (quint, J = 7.7 Hz, 2H), 2.03 (s, 3H), 2.63 (t, J = 7.3 Hz, 2H), 2.81 (t, J = 7.3 Hz, 2H), 2.91 (t, J = 7.7 Hz, 2H), 5.72 (s, 2H), 6.81 (d, J = 7.7 Hz 1H), 6.88 (s, 1H), 7.11 (d, J = 7.7 Hz 1H), 7.22 (d, J = 7.6 Hz 1H), 7.51 (ddd, J = 1.2 Hz, 7.7 Hz, 1H), 7.64 (ddd, J = 1.2 Hz, 7.9 Hz, 1H), 7.86 (d, J = 8.4 Hz, 1H), 8.96 (d, J = 8.7 Hz, 1H), 9.07 (s, 1H) ppm; ^{13}C NMR (75 MHz, $CDCl_3$, 298 K): δ = 13.9, 22.6, 27.3, 29.5, 29.7, 30.1, 44.7, 49.1, 118.0, 120.5, 121.9, 123.2, 125.5, 127.8, 127.9, 128.5, 128.9, 129.8, 130.9, 134.9, 136.0, 138.5, 142.7, 158.0, 207.4 ppm; HRMS (ESI): *m/z* calcd for $C_{25}H_{27}N_3O_2$: 402.2176 $[M+H]^+$; found: 402.2169.

Synthesis of **5**

(4-(3-((4-amino-2-butyl-1*H*-imidazo[4,5-*c*]quinolin-1-yl)methyl)phenyl)butan-2-one):

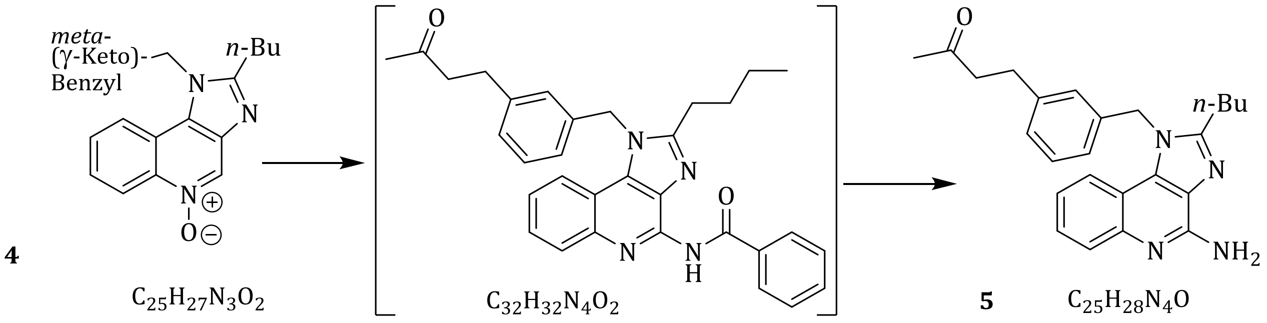

To a solution of **4** (226 mg, 0.56 mmol, 1 equiv.) in 25 ml dichloromethane, benzoyl isocyanate (1.68 mmol, 275 mg, 90%, 3 equiv.) was added. The mixture was heated at reflux under N_2 atmosphere for 70 h. The solvent was rotary evaporated to give (*N*-(2-butyl-1-(3-(3-oxobutyl)benzyl)-1*H*-imidazo[4,5-*c*]quinolin-4-yl)benzamide) as residue. (*N*-(2-butyl-1-(3-(3-oxobutyl)benzyl)-1*H*-imidazo[4,5-*c*]quinolin-4-yl)benzamide) was dissolved in 20 ml methanol, sodium methoxide (2.80 mmol, 154 mg, 5 equiv.) was added, and the mixture heated at reflux under N_2 atmosphere for 6 h. The mixture was concentrated under reduced pressure and the residue subjected to column chromatography (silica gel, DCM/MeOH = 25/1) for purification, which gave the title compound as pale orange foam (119 mg, 0.30 mmol, 53%).

^{1}H NMR (300 MHz, $CDCl_3$, 298 K): δ = 0.92 (t, J = 7.3 Hz, 3H), 1.43 (sext, J = 7.4 Hz, 2H), 1.79 (quint, J = 7.6 Hz, 2H), 2.04 (s, 3H), 2.63 (t, J = 7.3 Hz, 2H), 2.78–2.91 (m, 4H), 5.69 (s, 2H), 6.20 (br s, 2H), 6.84 (d, J = 7.6 Hz, 1H), 6.88 (s, 1H), 7.09–7.18 (m, 2H), 7.22 (d, J = 7.6 Hz, 1H), 7.45 (t, J = 7.8 Hz, 1H), 7.69 (d, J = 8.2 Hz, 1H), 7.83 (d, J = 8.4 Hz, 1H) ppm; ^{13}C NMR (75 MHz, $CDCl_3$, 298 K): δ = 13.9, 22.6, 27.3, 29.6, 30.0, 30.2, 44.9, 49.1, 114.6, 120.1, 123.1, 123.4, 125.2, 125.6, 126.4, 127.8, 127.9, 128.4, 129.7, 134.6, 135.3, 142.5, 150.8, 154.9, 207.5 ppm; HRMS (ESI): *m/z* calcd for $C_{25}H_{28}N_4O$: 401.2336 [M+H]$^+$; found: 401.2343.

Synthesis of $Cl_3PNSiMe_3$

Chlorophosphoranimine was synthesised similar to literature procedures [6, 7]. 26 g $LiN(SiMe_3)_2$ (155 mmol) were dissolved in 500 mL anhydrous diethyl ether under nitrogen at 0 °C and stirred for 30 min. 13.59 mL PCl_3 (155 mmol) were then added dropwise at 0 °C. The solution was allowed to warm to room temperature and stirred for 1 hour. After cooling to 0 °C again, 12.56 mL SO_2Cl_2 (155 mmol) were added and the mixture was stirred for another hour at 0 °C. Afterwards the reaction was filtered and the solvent removed under vacuum. The product was purified by vacuum distillation at 40-50 °C and 5 mbar to yield chlorophosphoranimine as colourless liquid. The product was stored under inert argon atmosphere at –35 °C.

Yield: 14 g (40%), ^{1}H NMR (300 MHz, $CDCl_3$, δ): 0.18 (s, 9H) ppm, ^{31}P NMR (121 MHz, $CDCl_3$, δ): - 54.3 ppm.

Synthesis of β-alanyl-boc-hydrazide

The boc-protected linker, β-alanyl-boc-hydrazide was synthesised according to literature procedures [11]. Z-β-Ala-OH (5.00 g, 22.4 mmol), boc-NH-NH_2 (2.96 g, 22.4 mmol) and N-(3-dimethylaminopropyl)-N´-ethylcarbodiimide hydrochloride (EDCI) (4.51 g, 23.51 mmol) were dissolved in 200 mL CH_2Cl_2 and stirred for 2 hours at room temperature. Afterwards the reaction mixture was extracted with 200 mL of 0.1 M acetic acid and the aqueous layer was extracted three times with 50 mL CH_2Cl_2. The organic layers were then combined and extracted twice with 200 mL 0.1 M acetic acid, twice with 200 mL of saturated aqueous sodium hydrogen carbonate and once with 200 mL H_2O. After drying over $MgSO_4$, the solvents were removed under vacuum and the product then further dried under high vacuum to yield Z-β-alanyl-boc-hydrazide as a white powder. The product (5.01 g, 14.86 mmol) was hydrogenated at 3 bar in 150 mL methanol with 10% Pd–C (0.3 g) for 24 hours. The reaction was filtered through Celite and rotary evaporated. The product was dried under high vacuum to yield β-alanyl-boc-hydrazide as a white foam.

Yield: 2.96 g (65%); FTIR (solid): ν_{max} = *3259br* (N–H), *2977w* (C–H), *1671s* (C=O); ^{1}H NMR (300 MHz, $CDCl_3$, δ): 1.46 (s, 9H), 2.50 (m, 2H), 3.13 (m, 2H), 5.30 (br, 4H) ppm.

Polymerisation procedure

The polymer was synthesised via the living cationic polymerisation of chlorophosphoranimine. In the glove box, initiator PCl_5 (14.85 mg, 0.07 mmol) and monomer $Cl_3PNSiMe_3$ (0.40 g, 1.78 mmol) were dissolved in CH_2Cl_2 (5 mL) at room temperature. The solution was stirred for 12 h and the solvent removed under vacuum. The resulting polydichlorophosphazene was then dissolved in anhydrous THF in an inert atmosphere. 0.8 equivalent of Jeffamine M-1000 1.426 g, 1.426 mmol) and NEt_3 (0.54 g, 5.35 mmol) were then added to the polymer solution and allowed to react for 24 hours. An excess of the hydrazide linker (2.2 eq, 0.797 g, 3.92 mmol) was then added to the reaction mixture and allowed to react for a further 24 hours. The solvent was then removed under vacuum and resulting polymer was purified by dialysis (12 kDa cut-off) for 48 hours against deionized H_2O followed by 48 hours against EtOH. The solvent was removed under a stream of nitrogen and the polymer was dried under vacuum to give a waxy solid. Yield: 1.6g (82%). ^{1}H NMR (300 MHz, $CDCl_3$, δ): 1.10 (br, 3H), 1, 41 (br, 9H) 3.35 (s, 3H), 3.62 (s, 82H) ppm, ^{31}P NMR (121 MHz, $CDCl_3$, δ): 0,90 ppm. SEC: M_n 9.1 KDa, Đ = 1.3

Conjugation of TLR agonists

A sample of the protected polymer was dissolved in a 2:1 CH_2Cl_2:CF_3COOH solution and stirred for 3 hours. The solvent was then removed under high vacuum. The deprotected polymer was dissolved in anhydrous methanol (10 mL) and one equivalent per hydrazide group of **5** was added. The mixture was stirred under reflux for 3 days. The product was then purified by dialysis against ethanol for 5 days. The amount of **5** covalently bound to the polymers was measured in acetonitrile by UV-Vis spectroscopy from the absorbance at 324 nm. Yield: 33 mg (28 %), ^{1}H NMR (300 MHz, $CDCl_3$, δ): 1.13 (br, 3H), 3.38 (s, 3H), 3.64 (s, 82H) ppm, ^{31}P NMR (121 MHz, $CDCl_3$, δ): 1.08 ppm. SEC: M_n 9.0 KDa, Đ = 1.3.

Release studies

The release of **5** from the polymer was analysed by HPLC. 5 mg of the polymer conjugates were dissolved in 1.5 ml of a 2/1 mixture of buffer/acetonitrile (v/v). Acetate buffer was used for the investigations at pH 5 and Tris buffer for pH 7.4 and filtered using a syringe filter. The amount of released drug was then investigated by HPLC measurements after certain times and the samples were stored at 37 °C between each measurement. UV detection was carried out at 260 nm and the amount of the released drug was estimated using a calibration curve for the free drug. At pH 5, approximately 50 % release of compound **5** was observed after 2.5 hours and full release was observed after 30 hours, whereas at pH 7.4 only approx. 50% release of **5** was observed during the whole period measured. As complete clearance of the macromolecular carrier is a stringent requirement for subsequent *in vivo* applications, the degradation profiles of the polymers were also investigated. The degradation studies of the conjugates at 37°C, pH 5 and 7 measured by size exclusion chromatography (SEC), showed that the polymers are stable over a short period of time in an aqueous environment but degrade significantly to small molecules under these simulated physiological conditions within 10 weeks (SI-6). These results are comparable to previous degradation studies of amino substituted poly(organo)phosphazenes but could be easily accelerated or decelerated as and when required [12].

Degradation studies

The conjugates (6 mg) were dissolved in aqueous solutions with pH 7 or pH 5 (4 ml) and incubated at 37 °C. Aliquot (0.8 mL) were then removed at regular time intervals diluted with toluene and the solvents were evaporated. The polymer was then dissolved in DMF, filtered through a 45 mm PTFE filter and analysed by SEC with RI detection.

Stimulation of murine splenocytes

Spleens isolated from C57BL/6-Tg(TcraTcrb)1100Mjb/J (OT-I) mice were smashed through a 70 µm cell strainer to collect single cells that further were fluorescently labeled with 7 µM CFSE (Sigma Aldrich). Afterwards 300.000 splenocytes were cultured in 200 µl complete medium [IMDM (Invitrogen) supplemented with 10% FBS (Sigma Aldrich), NEAA (Invitrogen), Penicillin Streptomycin (Invitrogen) and β-Mercaptoethanol (Sigma Aldrich)] in a 96-well plate and stimulated with 1 µg/ml ovalbumin derived SIINFEKL-peptide (Bachem) and with polymers loaded with **5**, R837 (InvivoGen) or R848 (Sigma Aldrich) at a final concentration of 0.1, 1 and 5 µM. All compounds were solved in DMSO (WAK Chemie Medical GmbH) at a stock concentration of 2 mM. The negative control cultures were

supplemented with 1 µg/ml SIINFEKL-peptide and the respective amount of DMSO alone. After 4 days cells were harvested and measured for proliferation in the CD8+ T cell compartment by flow cytometry using the following antibodies: CD3e APC, CD4 PerCP-Cy5.5, CD8a APC-eFlour®780, CD25 PE-Cy and vα2 TCR PE (all from eBioscience). Analysis was done with a BD LSR II Flow Cytometer (Becton Dickinson) and FlowJo Software 10.0.00002 (TreeStar).

The supernatants of splenocyte cultures were analyzed for IFN-γ by ELISA (eBioscience) according to the manufacturer's instructions. ELISAs were measured with an EnSpire Multimode Plate Reader (PerkinElmer).

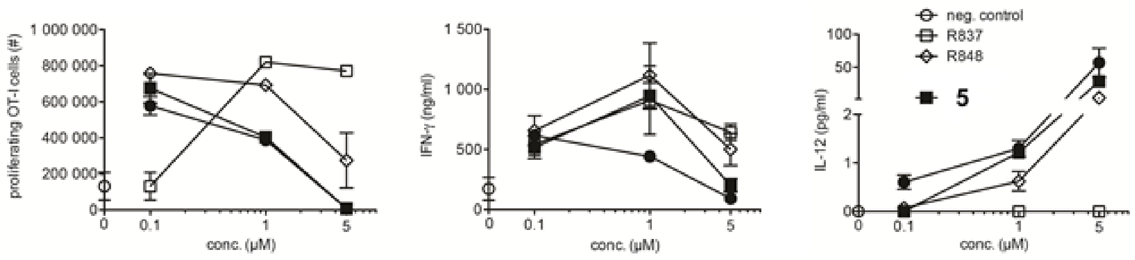

SI-1: Compound **5** induces T-cell proliferation and cytokine release. Splenocytes of transgenic OT-I mice were isolated, dyed with CFSE and stimulated for 4 days with SIINFEKL-peptide and the TLR-agonists R848 or R837 (positive controls) or **5**. DMSO was used as the negative control. On day 4, the T-cell proliferation was determined in the flow-cytometer. Cytokine secretion (IFNγ und IL-12) was analyzed via ELISA technique. (A) Proliferation of OT-I T-cells 4 days after the stimulation with 0.1 µM, 1 µM and 5 µM with **5**. IFNγ und IL-12 secretion levels are also shown. Polymer only and SIINFEKL peptide alone dissolved in DMSO served as negative controls.

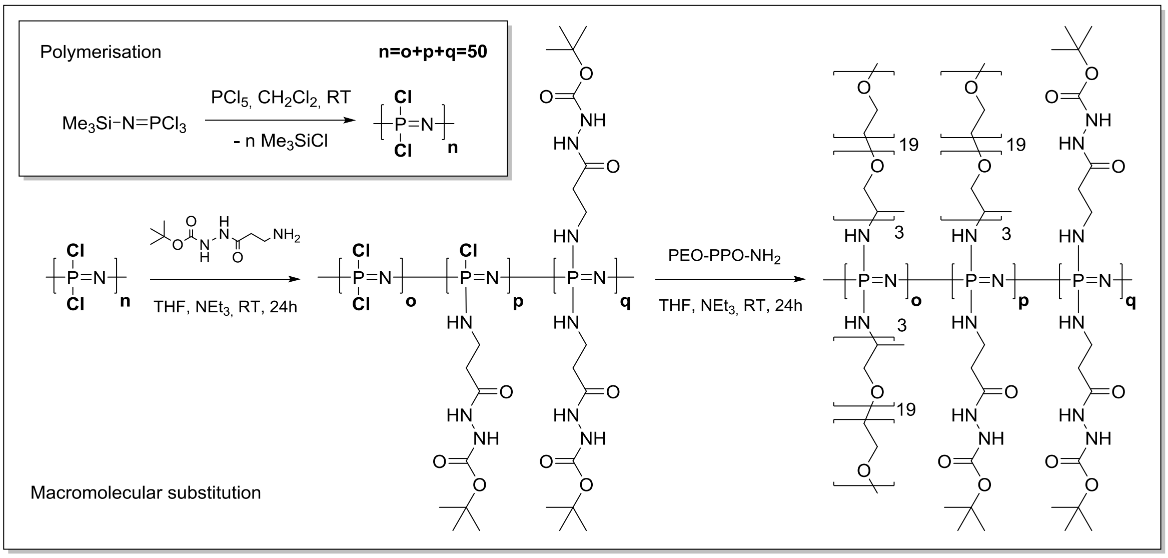

SI-2: Living polymerisation of polydichlorophosphazene and macromolecular substitution of the chlorine atoms by β-alanyl-boc-hydrazide and Jeffamine M-1000 (PEO-PPO-NH_2). The combinations of the two different side chains are expected to be statistically distributed.

The relative ratio of the β-alanyl-boc-hydrazide and the Jeffamine side chains was calculated by ^{1}H NMR, through integration of the Jeffamine protons (s, 3H) at 3.35 ppm versus those from the boc groups (s, 9H) of the hydrazide linker at 1.4 ppm. A ratio of 43 % β-alanyl-boc-hydrazide to 57 % Jeffamine M-1000 was obtained.

Table 1: Structural data for polymer 1-3

	drug	drug loading, wt%[a]	loaded linker, %[b]	PDI (SEC)[c]	M_n, kDa (S)[c]
Polymer		-	-	1.3	9
Conjugate	**5**	4.8	20,22	1.3	9.1

[a]Weight percent of total conjugate calculated from UV-Vis measurements. [b]Amount of hydrazide linker loaded with drug calculated from UV-Vis measurements. [c]Measured by SEC analysis and calibrated against linear polystyrene standards.

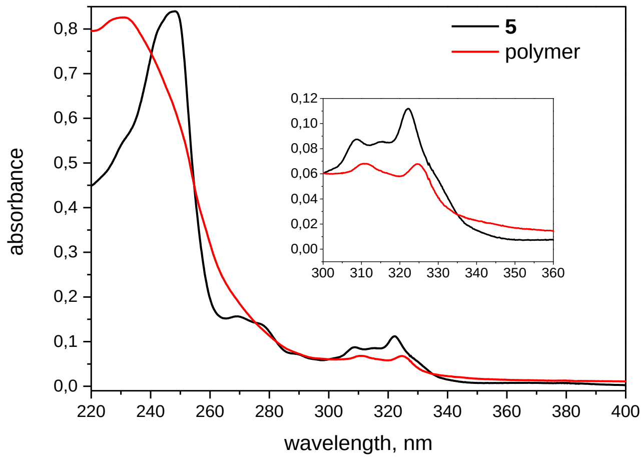

SI-3: UV-Vis spectra in acetonitrile of **5** (black), its conjugate (red)

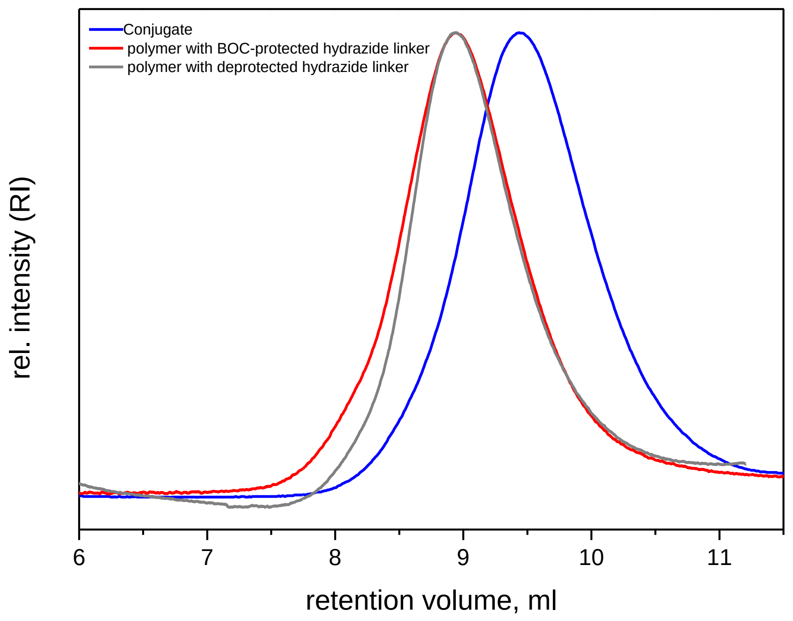

SI-4: SEC chromatograms of the polymer with boc-protected hydrazide linker (red), polymer with deprotected hydrazide linker (grey), and polymer with bound TLR agonist **5**.

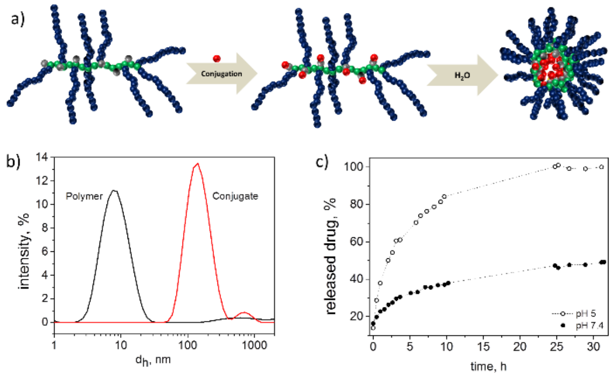

SI-5 a) Schematic representation of the intra- and intermolecular agglomeration and self-assembly of the polymers upon conjugation of the hydrophobic drug in aqueous solvents. b) Molecular size distribution by intensity as detected by dynamic light scattering for polymers 2–5 in phosphate buffer at pH 7.4 (polymer concentration 1 mg/mL, d_h - hydrodynamic diameter). c) Release of **5** from the conjugate at 37°C in acidic environment (acetate buffer, pH 5), and a neutral solution (pH 7.4, tris buffer). The amount of the released drug was estimated using a calibration curve for the free drug.

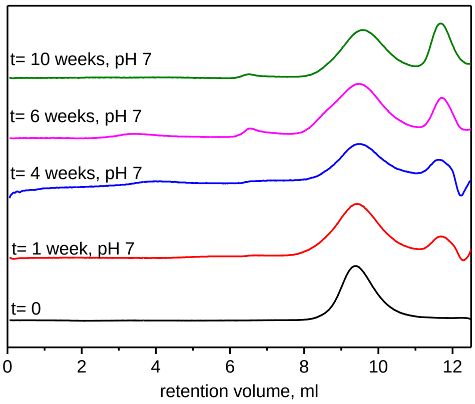

SI-6: SEC chromatograms showing the degradation of the conjugate at 37 °C in an aqueous solution at pH 5 and 7. Broadening and decrease in intensity and a shift to longer retention time of the conjugate peak and an increase in the peak of the cleaved polyalkylene oxide side chains are observed. A faster degradation is observed at pH 5 (above) than at pH 7 (below). A broadening and a shift to longer retention time of the polymer peak are observed. Moreover, a peak increasing over the time at a later retention volume was observed, attributed to the polyalkylene oxide side chains which are cleaved from the polymer.

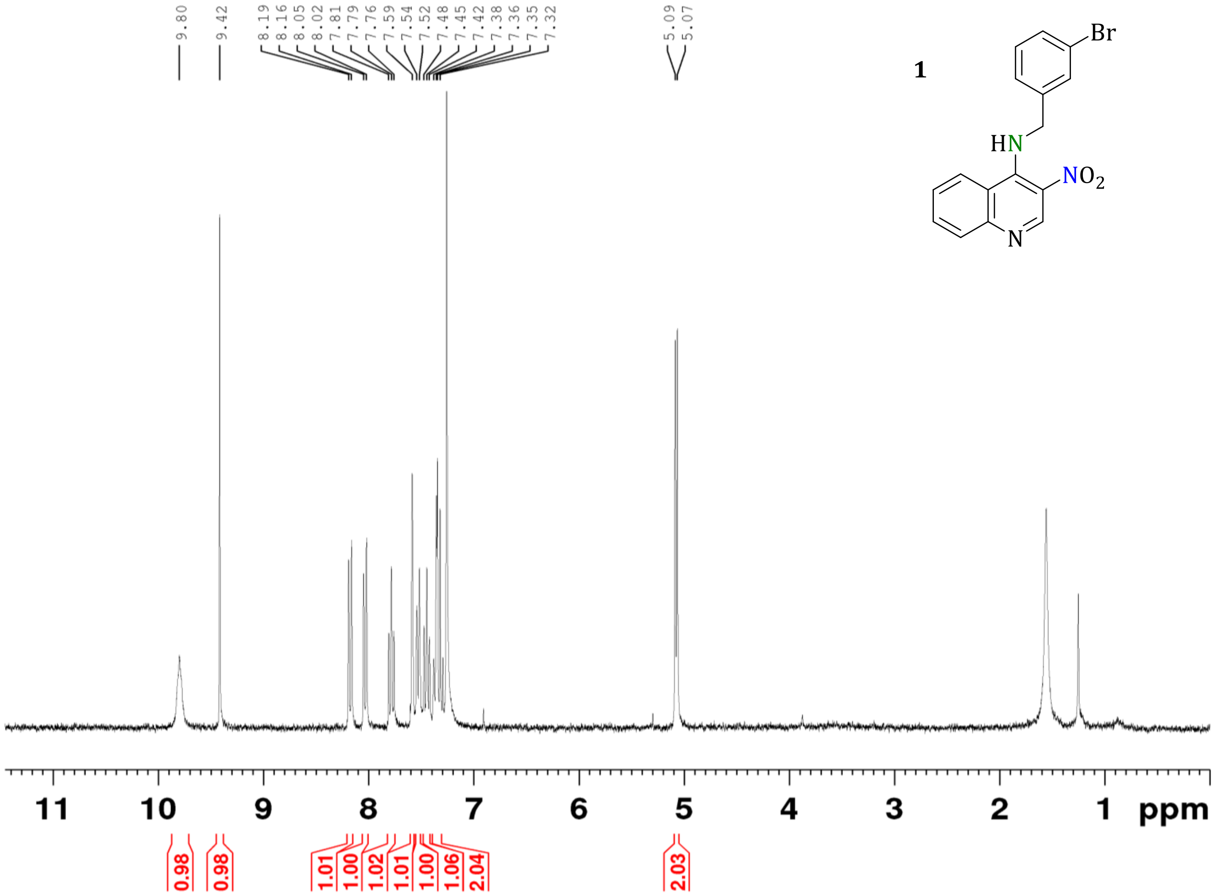

SI-7: ^{1}H NMR spectrum of **1** in $CDCl_3$

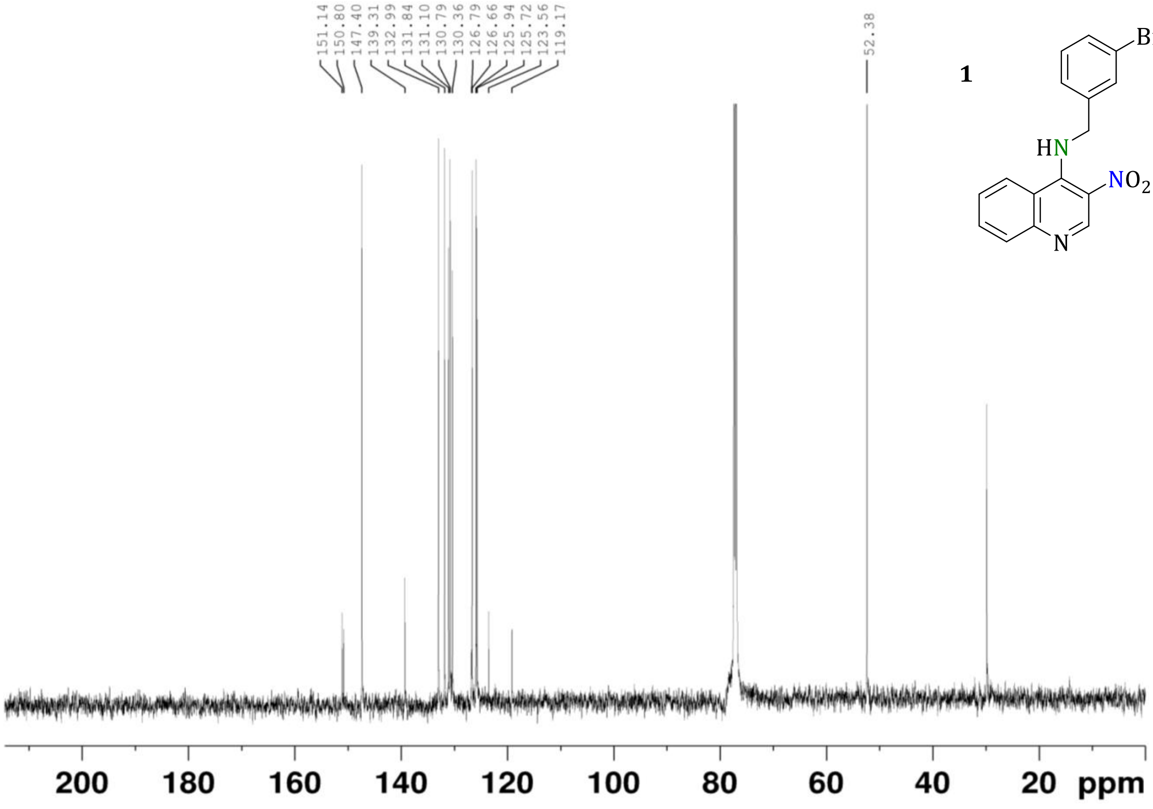

SI-8: ^{13}C NMR spectrum of **1** in $CDCl_3$

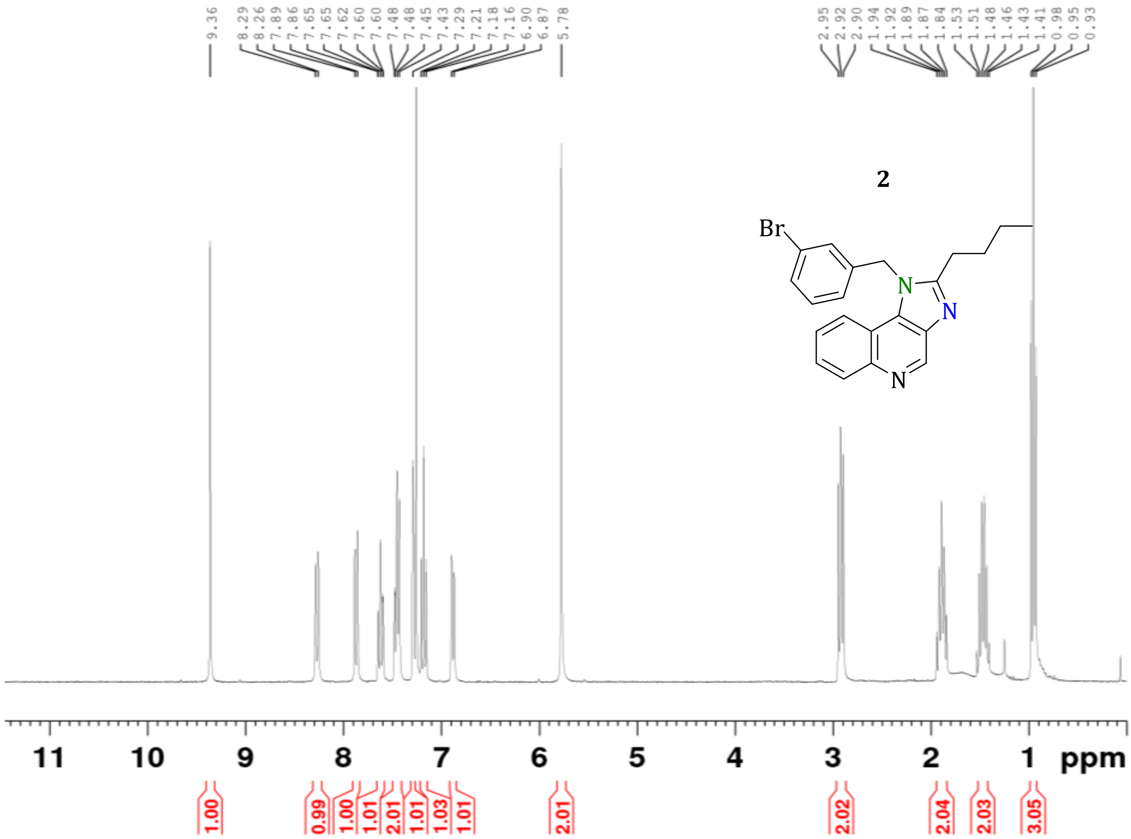

SI-9: ^{1}H NMR spectrum of **2** in $CDCl_3$

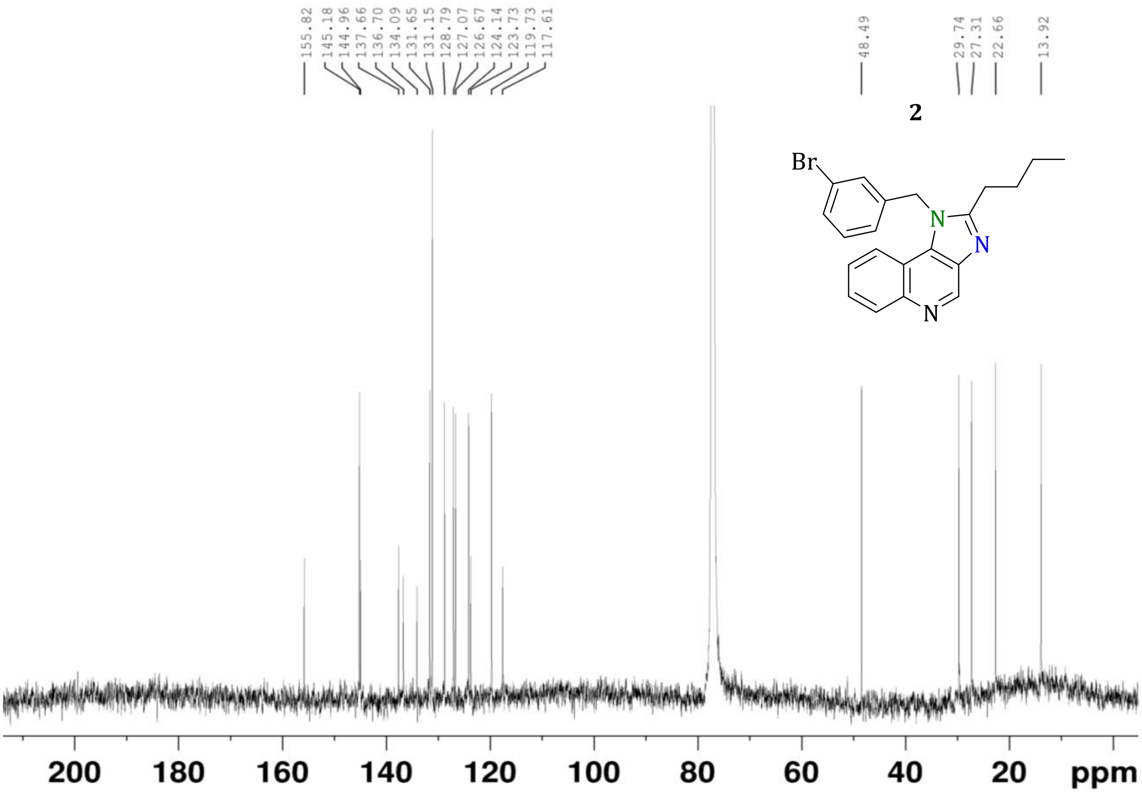

SI-10: ^{13}C NMR spectrum of **2** in $CDCl_3$

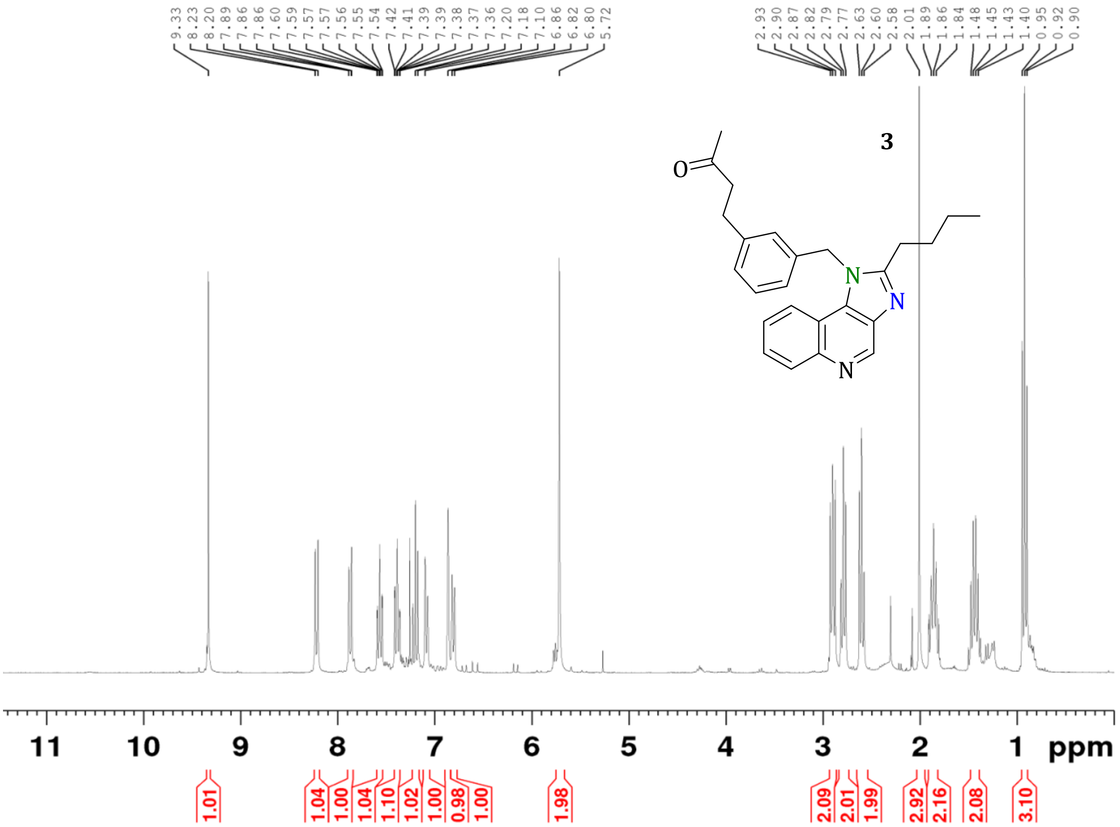

SI-11: ^{1}H NMR spectrum of **3** in $CDCl_3$

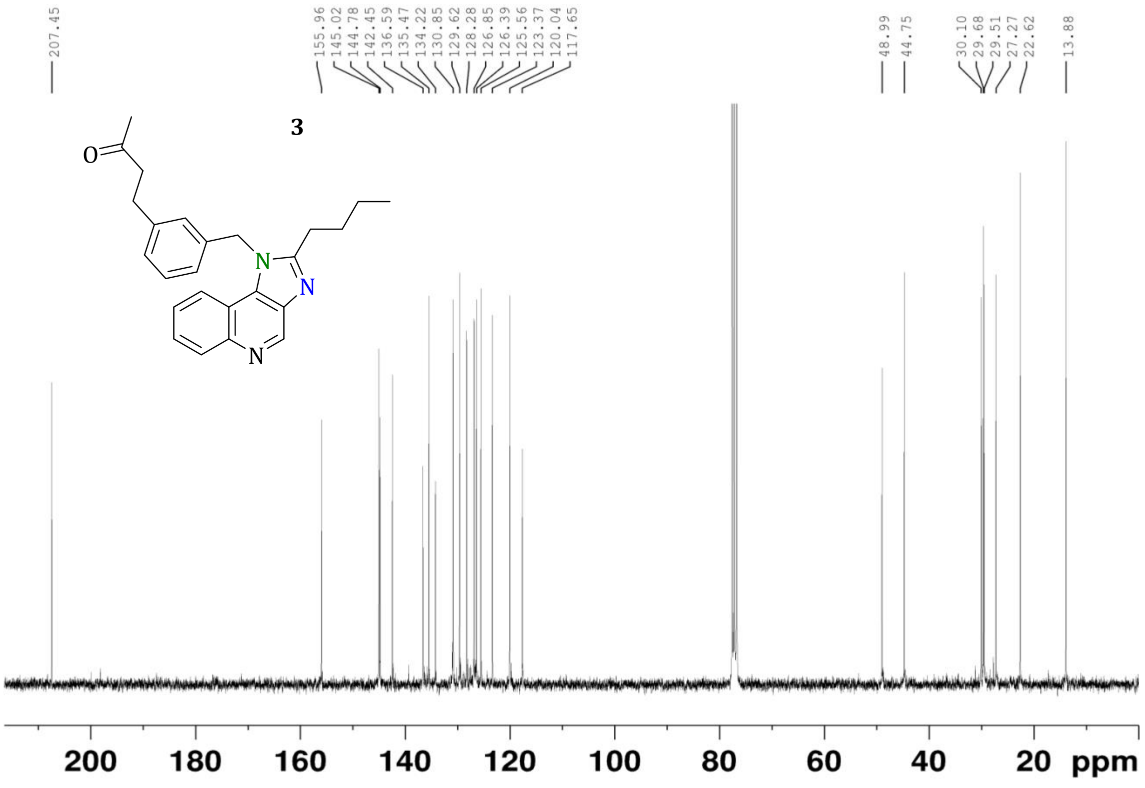

SI-12: ^{13}C NMR spectrum of **3** in $CDCl_3$

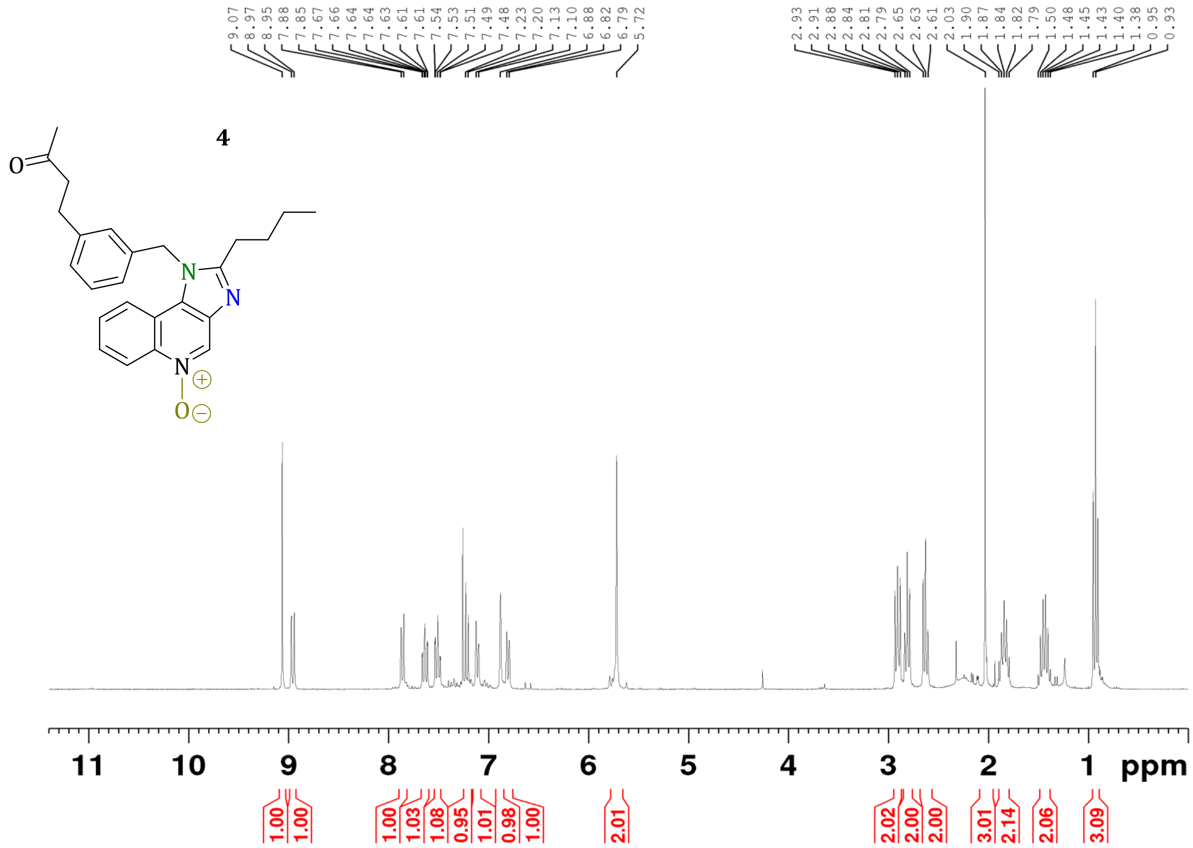

SI-13: ^{1}H NMR spectrum of **4** in $CDCl_3$

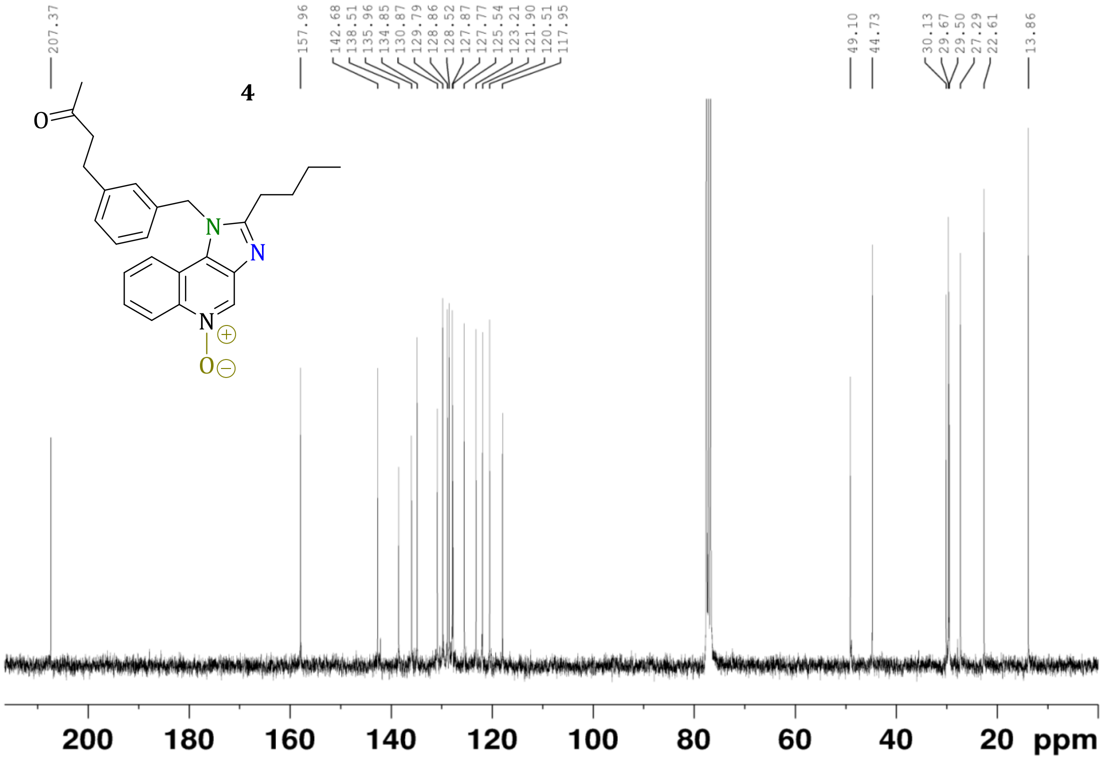

SI-14: ^{13}C NMR spectrum of **4** in $CDCl_3$

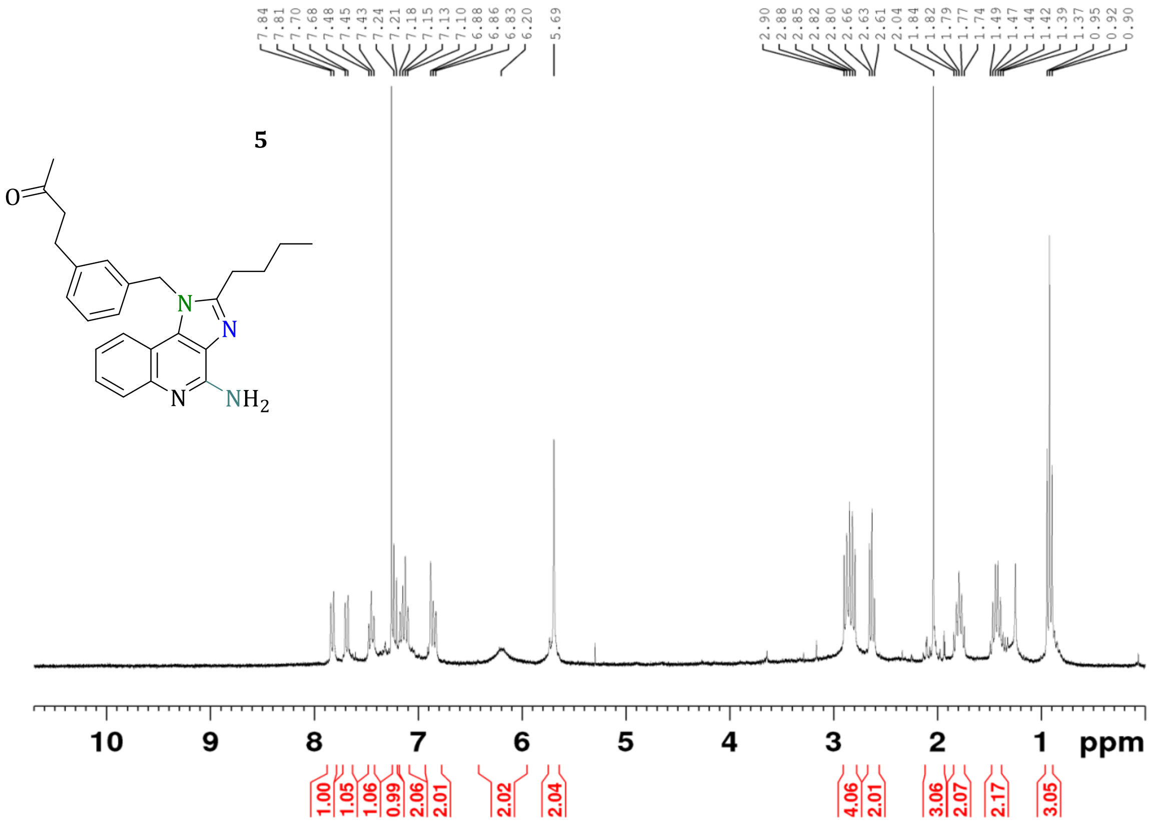

SI-15: ^{1}H NMR spectrum of **5** in $CDCl_3$

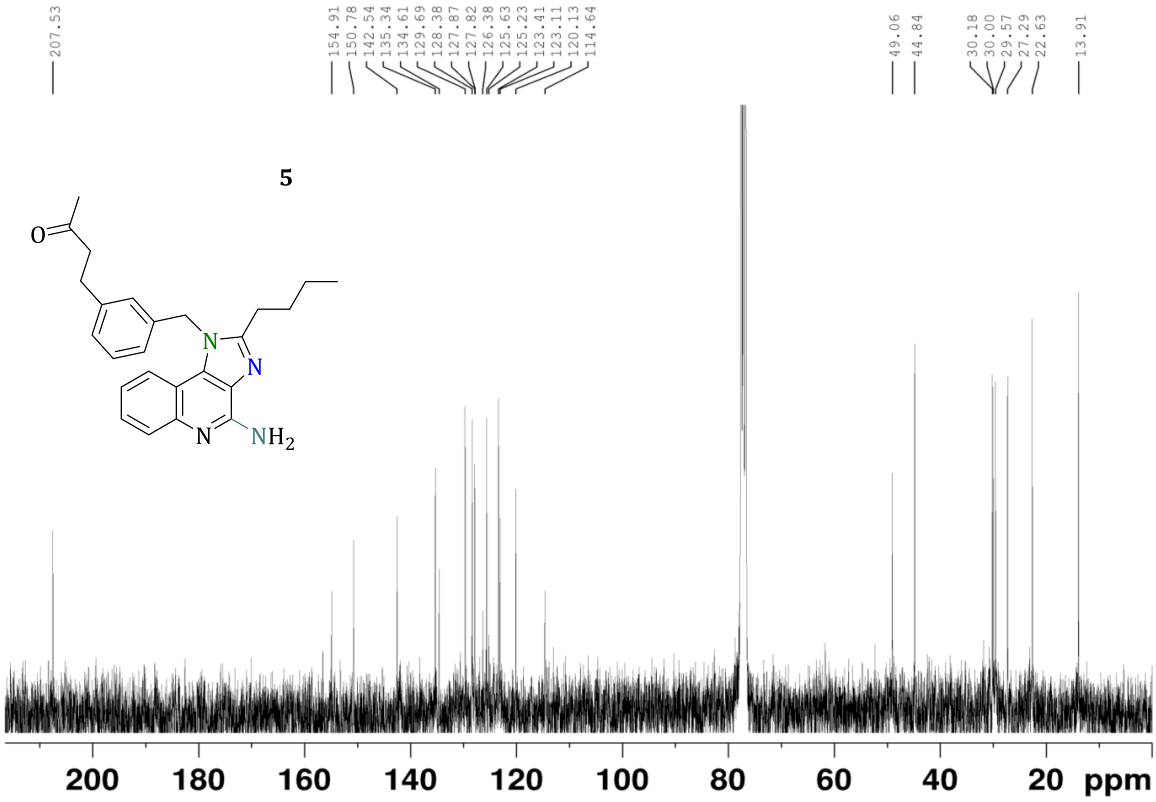

SI-16: ^{13}C NMR spectrum of **5** in $CDCl_3$

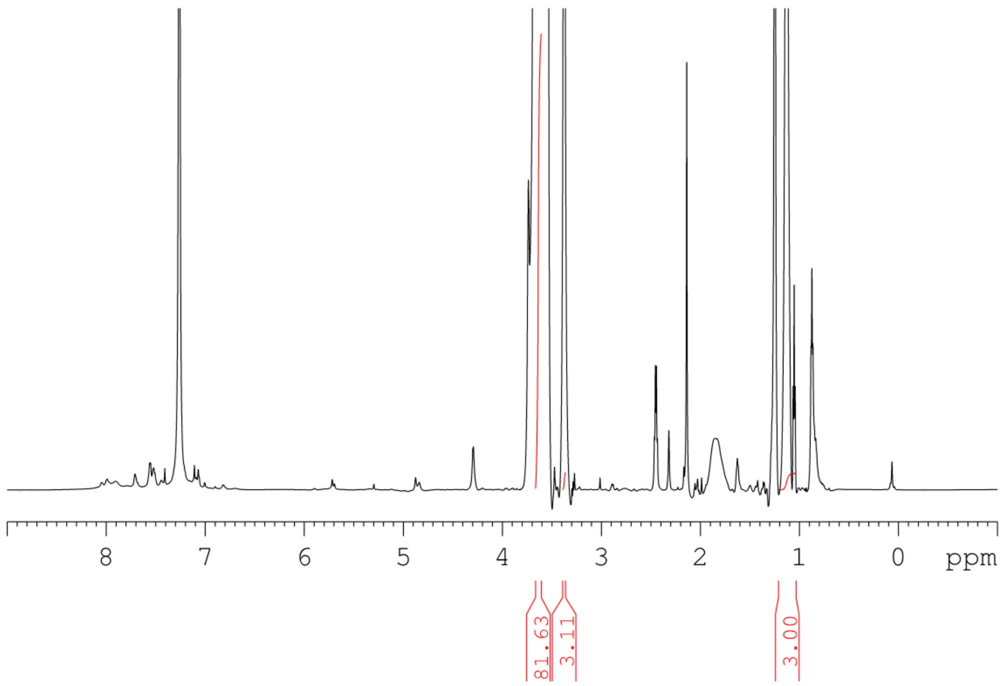

SI-17: ^{1}H NMR spectrum of the polymer conjugate in $CDCl_3$.

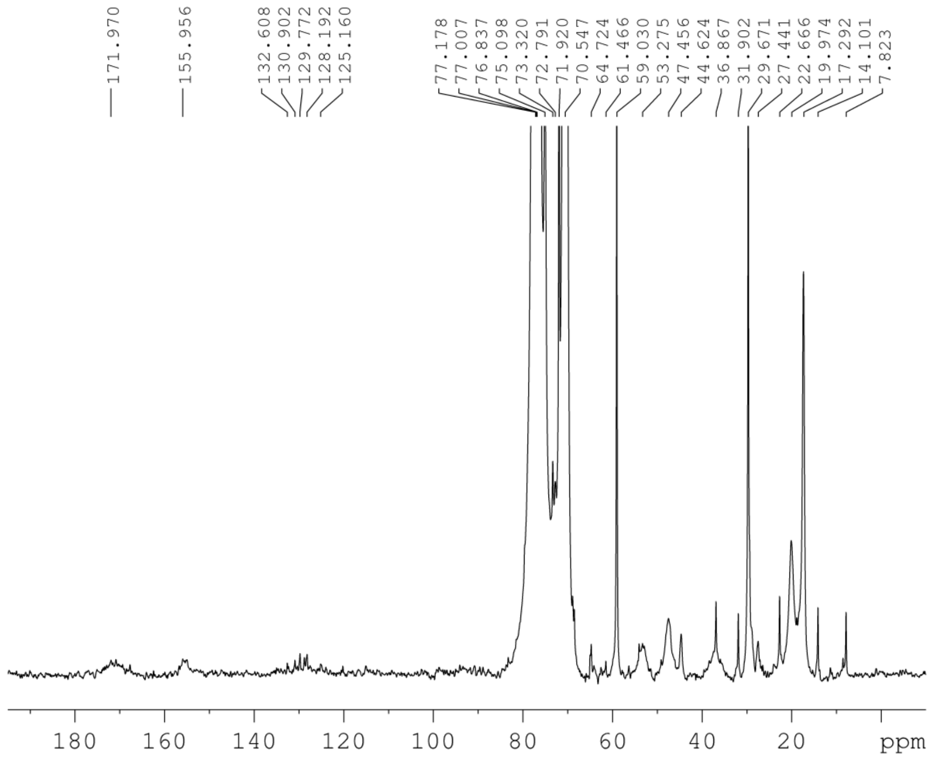

SI-18: ^{13}C NMR spectrum of the polymer conjugate in $CDCl_3$.

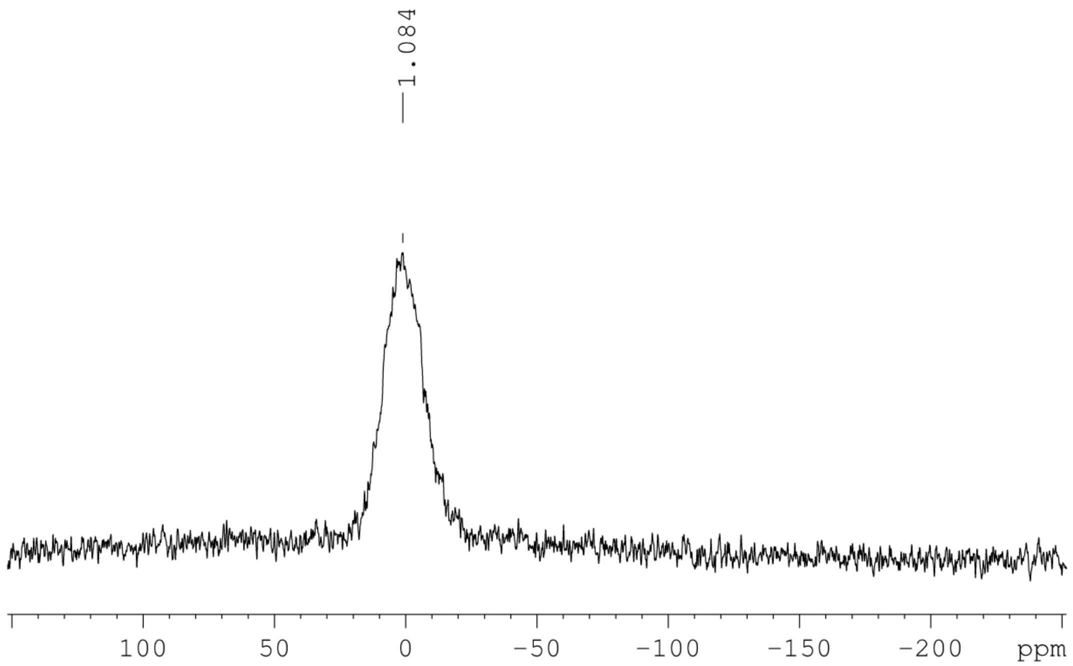

SI-19: ^{31}P NMR spectrum of the polymer conjugate in $CDCl_3$.

References

1. Ellett, F., Pase, L., Hayman, J.W., Andrianopoulos, A., and Lieschke, G.J. mpeg1 promoter transgenes direct macrophage-lineage expression in zebrafish. Blood **2011**, *117*(4): e49-e56
2. Kanther, M., Sun, X., Mühlbauer, M., Mackey, L.C., Flynn, E.J., Bagnat, M., Jobin, C., and Rawls, J.F. Microbial colonization induces dynamic temporal and spatial patterns of NF-kappaB activation in the zebrafish digestive tract. Gastroenterology, **2011**, *141*(1): 197-207.
3. van der Vaart, M., van Soest, J.J., Spaink, H.P., and Meijer, A.H. Functional analysis of a zebrafish myd88 mutant identifies key transcriptional components of the innate immune system. Dis Model Mech **2013**, *6*(3): 841-54.
4. Howe, K., et al., *The zebrafish reference genome sequence and its relationship to the human genome.* Nature, **2013,** *496*(7446): 498-503.
5. Distel, M., Köster R. W., In Vivo Time-Lapse Imaging of Zebrafish Embryonic Development, Cold Spring Harb Protoc; **2007**: doi:10.1101/pdb.prot4816.
6. S. Aichhorn, M. Himmelsbach, W. Schofberger, Org. Biomol. Chem. 2015, 13, 9373.
7. D. Yang, D. Fokas, J. Li, L. Yu, C. M. Baldino, Synthesis 2005, 2005, 47; b) E. J. Hanan, B. K. Chan, A. A. Estrada, D. G. Shore, J. P. Lyssikatos, Synlett 2010, 2010, 2759.
8. X. Han, J. Wu, Angewandte Chemie, International Edition 2013, 52, 4637
9. Wang, B., Rivard, E., Manners, I., *Inorg. Chem.*, **2002**, *41*: 1690-1691.
10. Paulsdorf, J., Burjanadze, M., Hagelschur, K., Wiemhöfer, H., *Solid State Ionics*, **2004**, *169*: 25-33.
11. King, H. D., Yurgaitis, D., Willner, D., Firestone, R. A., Yang, M. B., Lasch, S. J., Hellstrom, K. E., Trail P. A., *Bioconjugate Chem.*, **1999**, *10*: 279-288.
12. H. R. Krüger, I. Schütz, A. Justies, K. Licha, P. Welker, V. Haucke, M. Calderón, *Journal of Controlled Release* **2014**, *194*, 189.
